# Heat limits scale with metabolism in ectothermic animals

**DOI:** 10.1111/1365-2656.70042

**Published:** 2025-05-12

**Authors:** Nicholas L. Payne, Jacinta D. Kong, Andrew L. Jackson, Amanda E. Bates, Simon A. Morley, James A. Smith, Jean‐Francois Arnoldi

**Affiliations:** ^1^ Trinity College Dublin Dublin Ireland; ^2^ University of Victoria Victoria British Columbia Canada; ^3^ British Antarctic Survey Cambridge UK; ^4^ University of California Santa Cruz Santa Cruz California USA; ^5^ Centre National de la Recherche Scientifique Station d'Ecologie Théorique et Expérimentale de Moulis Moulis France

**Keywords:** acclimation, heat tolerance, physiology, scaling, thermal limits, warming temperatures

## Abstract

1. Ectotherms given time to acclimate to warmer environments, habitats or experimental treatments tend to tolerate higher maximum temperatures, but only slightly higher. This means warmer acclimated organisms live closer to their physiological temperature limits (their ‘critical temperatures’). The reason for this modest—and often highly variable—plasticity of heat limits is debated but raises concerns for resilience to future climate warming.
2. Experiments have shown heat tolerance is dependent not just on the magnitude of thermal stress but also on time via exposure duration. This implicates rate processes in the regulation of heat limits, yet few studies have explored this possibility. Invoking biological rates (such as metabolic rate) to explain the plasticity of critical temperatures is complicated by the need to account for temperature, time and the nonlinear dependence of rates on temperature.
3. We developed a new approach to explore whether incorporating estimated metabolic rate and its thermal scaling could explain the apparently modest and highly variable capacities of ectotherms to adjust their heat limits. To do this, we re‐evaluate a large thermal tolerance dataset for diverse ectothermic animals heated from different acclimation temperatures up to their critical temperature. By integrating temperature, time and the exponential relationship between temperature and metabolic rate, we compute a cumulative ‘metabolic currency’ that ectotherms expend (or accumulate) before reaching their heat limits. We then explore how this quantity varies for ectotherms acclimated to different temperatures.
4. Our ‘metabolic rescaling’ has a dramatic impact on explaining variation in heat limits, revealing that heating tolerance is effectively fixed within a species such that heat limits from any acclimation temperature can be predicted with remarkable accuracy by measuring heat limits at any other acclimation temperature. Heating rate also has a strong, consistent, influence.
5. Evidently, warmer‐acclimated organisms only marginally elevate their critical temperatures because they have a fixed amount of energy to spend during heating, and they spend it at a faster rate in warmer temperatures. This provides a very different perspective to leading explanations that organismal heat limits are constrained by hard physiological boundaries and instead encourages unification of thermal tolerance and metabolic scaling theory.

## Graphical abstract

We show that the extent to which ectotherms can increase their heat limits is strongly linked to how metabolic rate scales with temperature. This is a new perspective on what sets temperature limits in organisms, and will be important for understanding and predicting how different animals will respond to future warming.

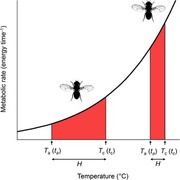

## 1 INTRODUCTION

The thermal tolerance limits of organisms are of increasing focus for understanding how rising temperatures and extreme heat events shape ecology and evolution (Sinclair et al., 2016) and for predicting organismal and community resilience to climate warming (Morley et al., 2019). Recent comparative analyses indicate that ectotherms have some capacity to increase their upper thermal tolerance limits through short‐term acclimation or adaptation, but that heat limits increase only marginally when an organism is acclimated to a higher temperature (acclimation response ratios tend to be <<1.0; Gunderson & Stillman, 2015; Pottier et al., 2022) or adapted to a warmer climate (Araujo et al., 2013; Gunderson & Stillman, 2015; Sunday et al., 2011). Phylogeny, experimental protocols and factors such as body size and ontogeny seem to explain some variation in thermal tolerance limits (Pottier et al., 2022; Rohr et al., 2018), but there remains considerable unexplained variation in the maximum temperature that an organism can tolerate (Gunderson & Stillman, 2015; Molina et al., 2024; Pottier et al., 2022; Rohr et al., 2018; Ruthsatz et al., 2024). The modest plasticity in heat limits, and uncertainty on why they vary between species and contexts, raises concerns about the ability of ectotherms to physiologically mediate the impact of future climate warming (Gunderson et al., 2017; Hoffmann et al., 2013), and of our ability to predict their resilience.

Experiments with ectotherms held under static temperature regimes have shown a strong dependency of time (e.g. exposure duration) on thermal tolerance; organisms held at higher temperatures tend to withstand the heat for shorter durations and in a way that can be quantified by exponential models if sufficient measurements are taken at various temperatures (i.e. thermal death time curves; Jørgensen et al., 2019; Rezende et al., 2014). Such models can produce accurate predictions of thermal tolerance under different conditions (Jørgensen et al., 2019, 2021; Rezende et al., 2020) and clearly show that time is an important factor for helping explain some variation in heat limits beyond that explained solely by temperature per se (Einum & Burton, 2023; Gunderson & Stillman, 2015; Pottier et al., 2022; Rohr et al., 2018). Nevertheless, thermal death time curves sometimes require making a large number of measurements of how temperature influences failure rates for a particular species, which means the approach can be experimentally onerous and difficult to generalise. In addition, the factors or mechanisms giving rise to the exponential influence of temperature on tolerance duration are unknown, although the time dependency implies biological rates must somehow be involved.

**FIGURE 1:**
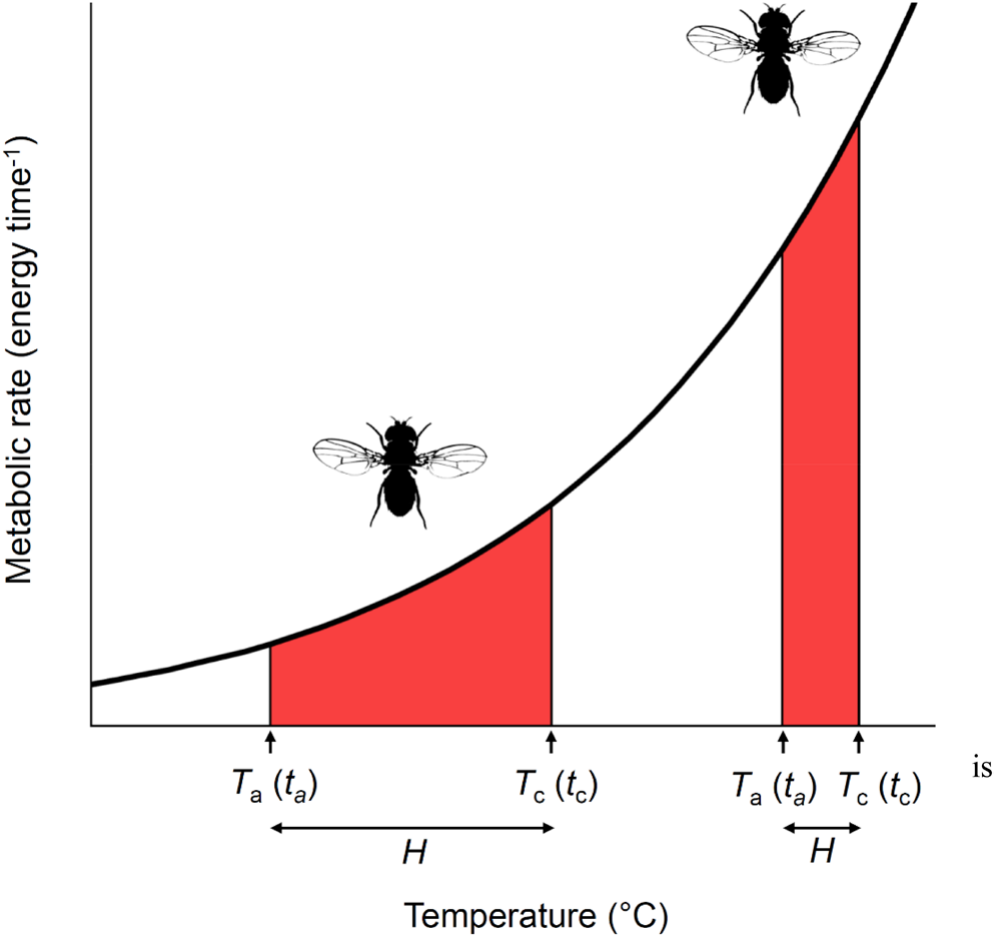
When an organism is heated, the temperature difference between the starting temperature *T*_a_ (at the time the heating experiment commences, *t*_a_) and temperature at the onset of collapse (*T*_c_, occurring at time *t*_c_) is denoted as the heating tolerance *H*. *H* is usually smaller for animals heated from a warmer starting temperate *T*_a_, so the time duration of heating tolerated also tends to be shorter than for cooler *T*_a_. Since most biological rates (such as metabolic rate) generally increase exponentially with temperature, we might expect the smaller *H* of warmer starting temperatures to arise because the total cumulative energy turnover (or damage accrued as a result; red shaded areas) is similar for an organism heated from cooler or warmer starting temperatures (i.e. in this figure, the red shaded polygons have the same area). Accordingly, we propose that rescaling to account for both the duration of heating (time) and the nonlinear influence of temperature on biological rates (e.g. thermodynamics) may be necessary to understand how *H* and *T*_c_ vary within and across organisms.

Biological rates tend to increase exponentially following a consistent Arrhenius form ~e^−0.6/*kT*^, where *k* is Boltzmann's constant and *T* is temperature (Brown et al., 2004). The ubiquity of this pattern across biology has given rise to the Metabolic Theory of Ecology, whereby the repeatable scaling of metabolic rate with temperature can be used as a helpful proxy of temperature scaling in many other biological or ecological rates (Dell et al., 2011). While the proximate mechanisms underlying failure of organism function at high temperature are debated, we might expect that heat failure rates scale in a similar fashion with temperature as does metabolic rate (and so many other biological processes; Dell et al., 2011). If they do, then rescaling thermal tolerances in line with metabolic rate could (a) explain a large amount of variation in heat limits of ectotherms, and (b) present a useful predictive model of thermal tolerance that requires very little a priori parametrisation. To test this hypothesis, we analysed a large dataset of ectothermic animals (316 species from 7 Phyla; Chordata, Arthropoda, Mollusca, Platyhelminthes, Echinodermata, Brachiopoda and Cnidaria) heated from different starting temperatures up to the temperature coinciding with physiological collapse. Using an integration approach following Arrhenius scaling of metabolic rate with temperature, we computed the equivalent metabolic currency that would be accumulated (such as heat damage) or expended (such as energy or substrate stores) during the heating event until collapse; we call this the ‘rescaled heating tolerance’ (it is conceptualised graphically in Figure 1). Our general prediction is that the quantity of this currency will be the same for a species whether heated from a low temperature or a high temperature up to its tolerance limit. We find remarkable accordance with our hypothesis, with data suggesting rescaled heating tolerance is effectively a fixed property of a species. Because of this consistency, heat limits under different thermal regimes can be predicted with surprising accuracy and precision by just measuring thermal tolerance from one starting temperature and adopting our rescaling approach. The findings build on recent studies showing cumulative impacts of thermal stress (Cook et al., 2024; Rezende & Carter, 2024), and represent a different perspective on the nature of heat limits, and provide a new empirical tool for predicting how heat limits will vary under different thermal regimes.

## 2 MATERIALS AND METHODS

### 2.1 Heating tolerance and heating duration

A common experimental procedure for determining ectotherm temperature limits is to heat an organism from a starting temperature (we call this the acclimation temperature, *T*_a_ in Celsius, given our underlying dataset, noting that some studies denote starting temperatures differently to those of acclimation) up to the temperature coinciding with physiological collapse; the critical temperature *T*_c_ in Celsius (sometimes called the ‘upper thermal tolerance limit’). Under such experimental procedures, we define the temperature range spanning a heating event as the heating tolerance, *H* (units of°C; Figure 1),

(1) $$H=T_{c}-T_{a}$$

Because such a heating event involves an increase in time as well as temperature, we can also denote an elapsed duration of the heating event that corresponds to the heating tolerance (Figure 1).

(2) $$\mathrm{\Delta t}=t_{c}-t_{a}$$

where Δ*t* represents the duration of the heating event that starts at the commencement of the temperature increase (*t*_a_; e.g. at 0 min) and ends at the time onset of physiological collapse that signals the end of the ramping assay at *T*_c_ (*t*_c_). Thus, these times correspond to a temperature in degrees Celsius (i.e. *t*_a_ at acclimation temperature and *t*_c_ at upper thermal tolerance).

**FIGURE 2:**
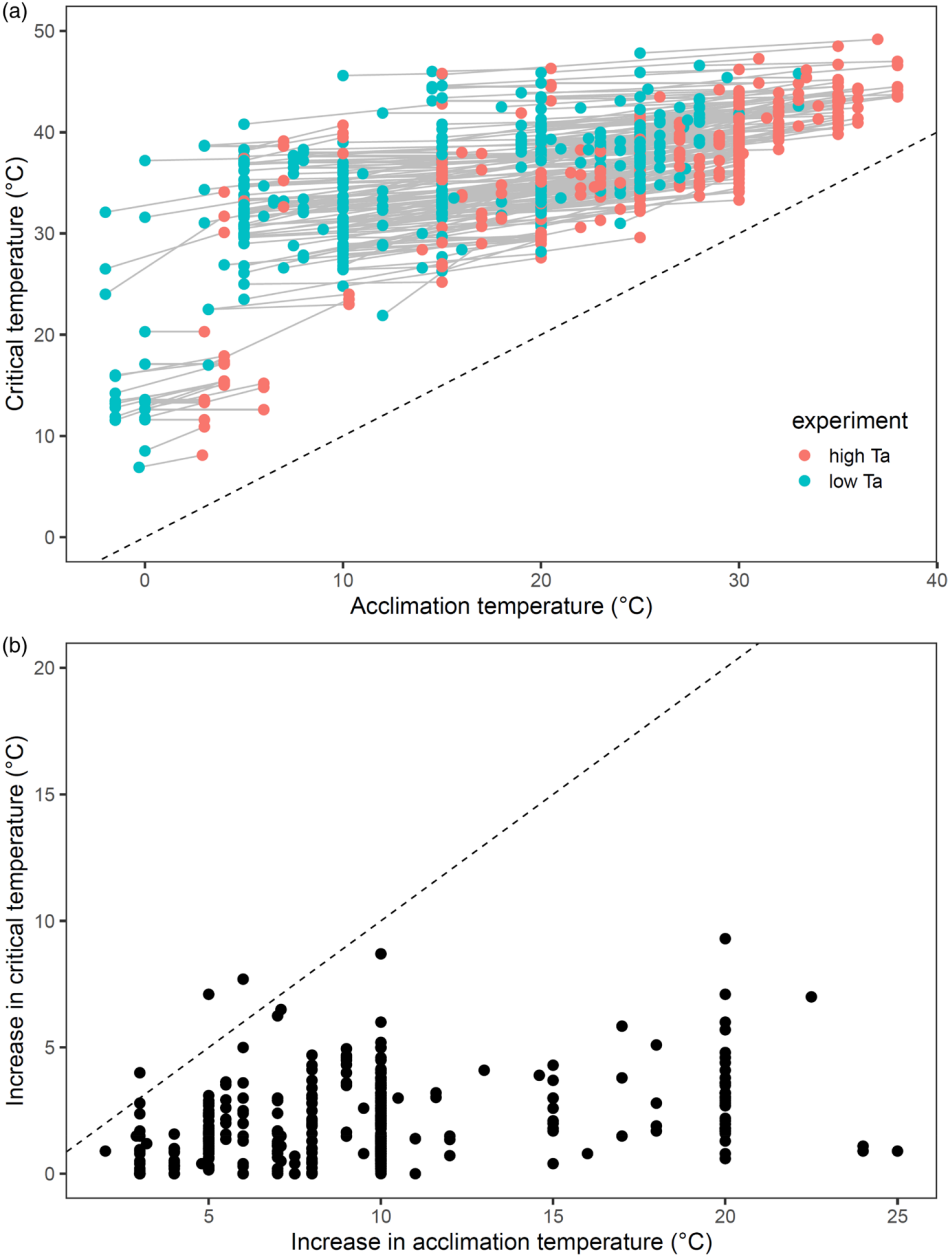
Thermal tolerance data used in this study. Panel (a) shows critical temperatures (*T*_c_) for ectotherms heated from two different acclimation temperatures (*T*_a_), with grey lines delineating the increase in *T*_a_ and *T*_c_ for a particular species. Panel (b) shows that an increase in *T*_a_ generally leads to a much smaller increase in corresponding *T*_c_ (most data fall well below the 1:1 unity line, which is dashed in both panels).

To test our hypothesis, we reanalysed a published dataset of heating tolerances where each ectotherm species was heated from two different starting temperatures (*T*_a_) up to the respective *T*_c_, having first been given hours to weeks to acclimate to those *T*_a_ (see Morley et al., 2018, 2019 for details). The two different acclimation temperatures per species were on average ~10°C apart (Supplementary Material). Plotting the raw data shows that an increase in *T*_a_—both between and within species—tends to lead to an increase in *T*_c_, but of a smaller magnitude than the increase in *T*_a_ (Figure 2). Since we used already published data, our study did not require ethical approval for the use of animals in research.

### 2.2 Estimating total energy turnover during a heating event

During a heating event from *T*_a_ to *T*_c_ (from time *t*_a_ to time *t*_c_), we would expect the rate of biological processes (such as metabolic rate) to increase with temperature according to

(3) $$e^{-0.66/\mathrm{kT}}\approx e^{\beta \tau }$$

where *T* is in Kelvin, *β* = 0.66/*kT*_K_ is a dimensionless constant constructed from the organism‐level activation energy that describes the temperature dependence of most biological rates (Dell et al., 2011), Boltzmann's constant *k* and the normalisation constant *T*_K_ (=273.15) is a reference temperature (equivalent to approximately 0°C) that simplifies comparisons across species and minimises the influence of extreme observations in the wide range of acclimation temperatures, heating rates and thermal tolerances reported in our dataset (Figure 2). The normalisation step converts the Arrhenius form (left side) of Equation 3 into a standard exponential function (right side) which is analytically integrable unlike inverse temperature. We used normalised temperature scales (τ = *T°*/*T*_K_ = normalised temperature and has no units, with *T°* in Celsius) for our subsequent comparisons, noting that normalised temperatures can be rescaled back into degrees Celsius by *T*° = *τ* × *T*_K_. A full explanation of our derivation of the exponential approximation of the Arrhenius form (i.e. Equation 3) can be found in Supplementary File S2.

We selected the value of activation energy a priori to define *β*, and changing this value (*E* = 0.66 eV) within realistic bounds does not meaningfully change our subsequent results (Figure S1).

Because biological rates proceed faster at higher temperatures (Equation 3), we can rescale the time elapsed between the start of the ramping assay (*t*_a_) and the end of the ramping assay (*t*_c_) as an integral of the (dimensionless) biological rate–temperature relationship $e^{\beta \tau \left(t\right)}$ over time:

(4) $$\Delta t_{r}=\int_{t_{a}}^{t_{c}}e^{\beta \tau \left(t\right)}\mathrm{dt}$$

This metric is the biological rate‐corrected elapsed duration of the ramping assay (we call this ‘rescaled heating tolerance’); it could reasonably be considered the *effective* duration of a heating event experienced by an organism, given that an organism's processes proceed faster at higher temperatures. We introduce subscript r at this point to denote variables that have been corrected for the exponential increase in biological rates with temperature (Table 1).

**TABLE 1:** Main mathematical notation and description used in this analysis.

Symbol	Description
*H*	Heating tolerance; range of temperatures between acclimation temperature (*T*_a_) and upper thermal tolerance (*T*_c_)
Δ*t*	Elapsed duration of a heating assay between the start (*t*_a_) and end (*t*_c_) time points
*r*	Variables rescaled by the temperature dependence of biological rates
*a*	Variables referring to acclimation or starting temperature of a heating assay
*c*	Variables referring to the upper thermal tolerance limit of a heating assay
*T*	Temperature in degrees Kelvin
*T°*	Temperature in degrees Celsius
*T*_K_	Reference temperature taking the value 273.15 K
*τ*	Normalised temperature scale centred around *T*_K_
*λ*	Normalised heating rate centred around *T*_K_
*γ*	Scaling exponent for the effect of heating rate Δ*t*_r_ on rescaled heating duration

Since heating experiments heat organisms at a variety of rates, we can also incorporate heating rate to estimate the total cumulative quantity of a biological rate (e.g. total metabolism) transformed during heating. If heating rate (expressed on the normalised temperature scale; *λ* = heating rate (°C time^−1^)/*T*_K_; units: time^−1^) is constant during the heating experiment, time and temperature become equivalent variables (since *dτ* = *λdt*) and the integral in Equation 4 can be simplified, with *τ*(*t*) becoming a constant *τ* in the exponent, and 1/*λ* moving outside the integral as a factor:

(5) $${\Delta t}_{r}=\frac{1}{\lambda }\int_{\tau_{a}}^{\tau_{c}}e^{\beta \tau }\mathrm{d\tau}$$

Equation 5 rescales temperatures, times and heating rates to estimate the total quantity of metabolism (or other biological rates that scale with temperature ~ $e^{-0.66/\mathrm{kT}}$) expended during a heating event from the starting temperature to the point of physiological collapse.

Our paired experimental data (a low and high *T*_a_ and *T*_c_ for each species) were heated at constant rates within species, so we rescaled our heating tolerance data (i.e. the Δ*t*_r_ metric) using Equation 4. We then compared rescaled heating tolerances for a species at its low *T*_a_ to its rescaled heating tolerance at its high *T*_a_. Our prediction is that unscaled heating tolerances will be poorly correlated between low and high *T*_a_ treatments (as they seem to be; Figure 2a), but that our rescaling procedure produces strong concordance in a species' heating tolerance across different *T*_a_ (a useful conceptualisation of this prediction could be that the red polygons in Figure 1 have the same area).

If it is indeed the case that our rescaling procedure effectively corrects unscaled heating tolerances such that the Δ*t*_r_ metric is ~ the same for a species heated from various *T*_a_s, then quantifying Δ*t*_r_ for just one *T*_a_ treatment will enable accurate prediction of a species' unscaled heating tolerance (and its *T*_c_) when heating starts from any other *T*_a_. To estimate heating tolerance at any *T*_a_ based on measured heating tolerance at any other *T*_a_, we use the form

(6) $$\Delta \tau \left(\tau_{a2}\right)=f_{\beta }\left(e^{\beta \left(\tau_{a1}-\tau_{a2}\right)}g_{\beta }\left(\Delta \tau \left(\tau_{a1}\right)\right)\right)$$

where Δ*τ*(*τ*_a2_) is the unknown heating tolerance starting from the unmeasured acclimation temperature, *τ*_a2_, estimated from a known heating tolerance, Δ*τ*, starting from an acclimation temperature which was measured in an experiment, *τ*_a1_. Equation 6 includes new simplifying expressions $f_{\beta }$ and $g_{\beta }$, which are defined and explained in the Supplementary Material. To test the utility of our Δ*t*_r_ metric to predict heating tolerance of a species at a new temperature based on measured heating tolerance at another temperature (i.e. a different starting *T*_a_), we computed Δ*t*_r_ for one *T*_a_ of all our data, then used equation 6 to predict what heating tolerance should be for the other *T*_a_ treatment for each species. We then compared predicted to observed heating tolerances.

It has long been recognised that heating rate influences thermal tolerance limits of organisms (Terblanche et al., 2007), so we also considered a second dataset of *T*_c_ from 5 phyla (Morley et al., 2016) where 37 species had heating tolerance (*H*) measured under multiple different constant heating rates but starting from the same acclimation temperature (Supplementary Material). As a secondary objective of our study, we undertook a series of analyses of how heating rate influences rescaled heating tolerance in this second dataset, as described in the Supplementary Material.

## 3 RESULTS

**FIGURE 3:**
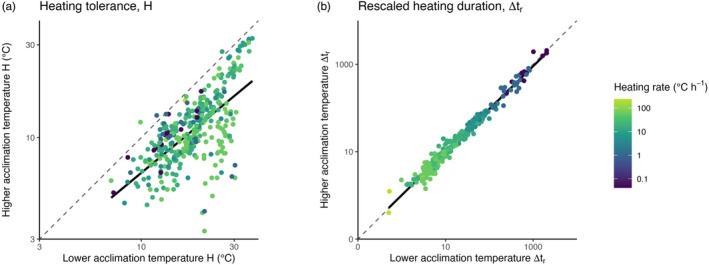
Large variation and poor concordance in heating tolerance (*H*) is seen for species acclimated to low and high temperatures on linear temperature scales (a), but accounting for the exponential relationship between temperature and biological rates (rescaled heating tolerance Δ*t*_r_) removes a lot of this variation and closely aligns heating tolerance between low and high acclimation temperatures (b). Points should fall along the identity line (dashed line) if rescaled heating tolerances are the same at both acclimation temperatures and compared with the solid line of an ordinary least‐squares regression of the observations. Heating rates (colours) are constant and identical for paired observations. Axes are Log_10_ transformed.

The data used in our study are displayed in Figure 2. At a course scale, *T*_c_ appears to weakly increase as *T*_a_ increases both between and within species (Figure 2a), but a particular increase in *T*_a_ corresponds to a far smaller increase in corresponding *T*_c_ (i.e. almost all data sit well below the unity line in Figure 2b). Viewing those data in another way, the heating tolerance *H* (*T*_c_ − *T*_a_) of a species at low *T*_a_ is correlated with its *H* at the high *T*_a_, but that correlation is weak and variable, and *H* is almost always a lot lower for the warmer *T*_a_ than *H* at the cooler *T*_a_ (Figure 3a). Taken together, the data show that ectotherms acclimated to higher temperatures only increase their maximum temperature limit marginally, and that heating tolerance of a species at one acclimation temperature is a poor predictor of its heating tolerance at other temperatures.

**FIGURE 4:**
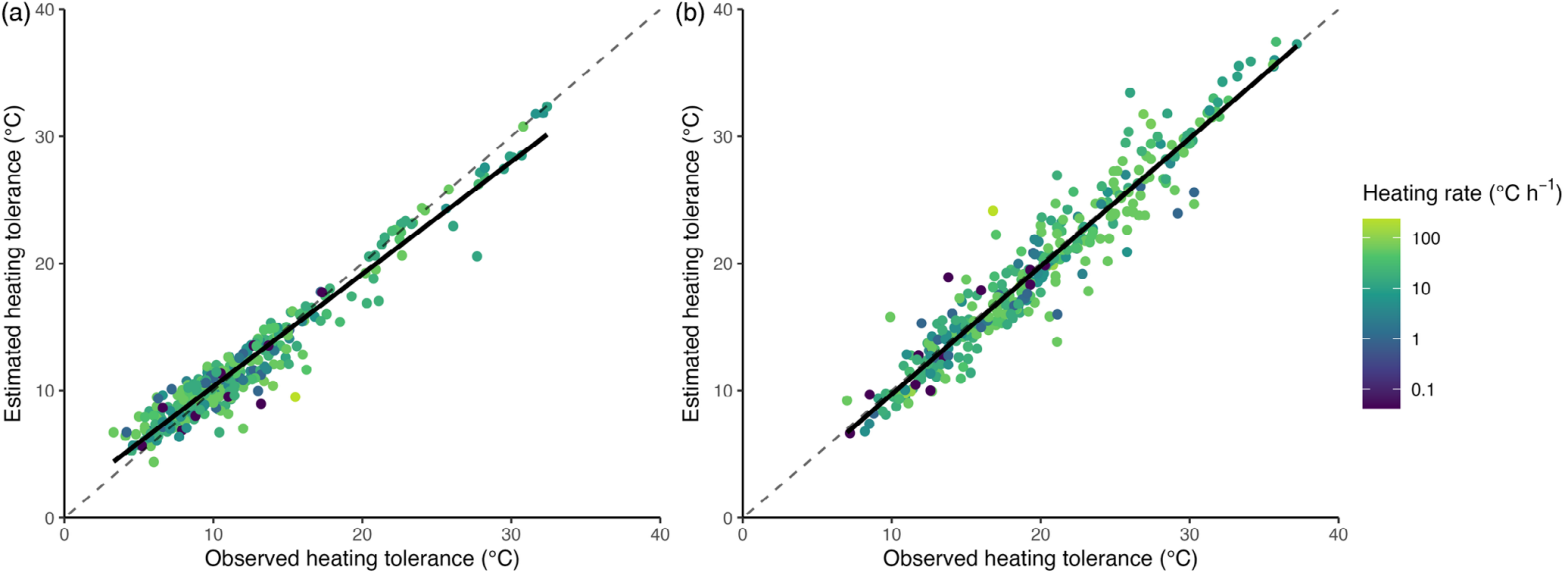
Observed versus estimated heating tolerance (*H*) at (a) the higher acclimation temperature estimated from heating tolerance at the lower acclimation temperature, and (b) the lower acclimation temperature estimated from heating tolerance at the higher acclimation temperature. Estimations were made by rescaling heating tolerance at one acclimation temperature, and assuming the rescaled tolerance is the same for the other acclimation temperature. Points should fall along the identity line (dashed line) if rescaled heating tolerances are the same at both acclimation temperatures (and compared with the solid line of an ordinary least‐squares regression). Heating rates (colours) are constant and identical for paired observations.

However, when we adopt our rescaling approach to account for the nonlinear temperature dependence of biological rates, the interpretations change dramatically. Rescaled heating tolerance, Δ*t*_r_, was strikingly conserved within a species acclimated to very different temperatures, with all data clustering along the identity line when comparing rescaled heating tolerance for heating assays starting from lower versus higher acclimation temperature (Figure 3b). This means that rescaled heating tolerance at one temperature is a very good predictor of rescaled heating tolerance at another temperature. Indeed, our use of Equation 6 to predict heating tolerance at one acclimation temperature by computing Δ*t*_r_ at another acclimation temperature (and assuming it is a fixed quantity for a species) provides excellent predictions of heating tolerances (regression slope for estimating higher acclimation temperature from low acclimation temperature = 0.88, *R* ^2^ = 94%; regression slope for estimating lower acclimation temperature from higher acclimation temperature = 1.01, *R* ^2^ = 92%, Figure 4). It is worth emphasising here that the predictions in Figure 4 are not formulated by incorporating any phenomenological expression of how upper thermal tolerance varies with any other factor; they are made purely by measuring upper thermal tolerance *T*_c_ for just one acclimation temperature and by assuming a priori and then incorporating the scaling of the nonlinear temperature dependence of biological rates (~e^−0.66/*kT*^).

The supplementary exploration of heating rate showed that rescaled heating tolerance Δ*t*_r_ exhibited a strong dependency on heating rate within and between species, with faster heating reducing the rescaled heating tolerance estimated during heating up to the upper thermal tolerance limit (Figure S4A; data fall further below the 1:1 line towards larger *x*‐values). This influence of heating rate is well described by a power function of the form Δ*t*_r_(*λ*) ~ *cλ* ^−*γ*^ with an interspecific scaling exponent of *γ* ~ 0.76, albeit with interspecific variation (Figure S4B; individually coloured lines). Fitting of random slopes for the different species lowered the exponent slightly to *γ* ~ 0.71. Species‐specific scaling exponents range between −0.06 and −1.05. Using species‐specific values of *γ* in this phenomenological scaling expression to estimate heating tolerance from a known heating tolerance at another acclimation temperature produced good approximations between estimated heating tolerance and observed values of heating tolerance for all pairwise combinations of heating rate (Figure S4C; data for estimated vs. measured heating tolerance clustered along the identity line; *R* ^2^ = 87.9%). Using the interspecific scaling exponent of 0.76 for *γ* produced less accurate estimations of heating tolerance than using species‐specific values (Figure S4D; *R* ^2^ = 54.51%).

**FIGURE 5:**
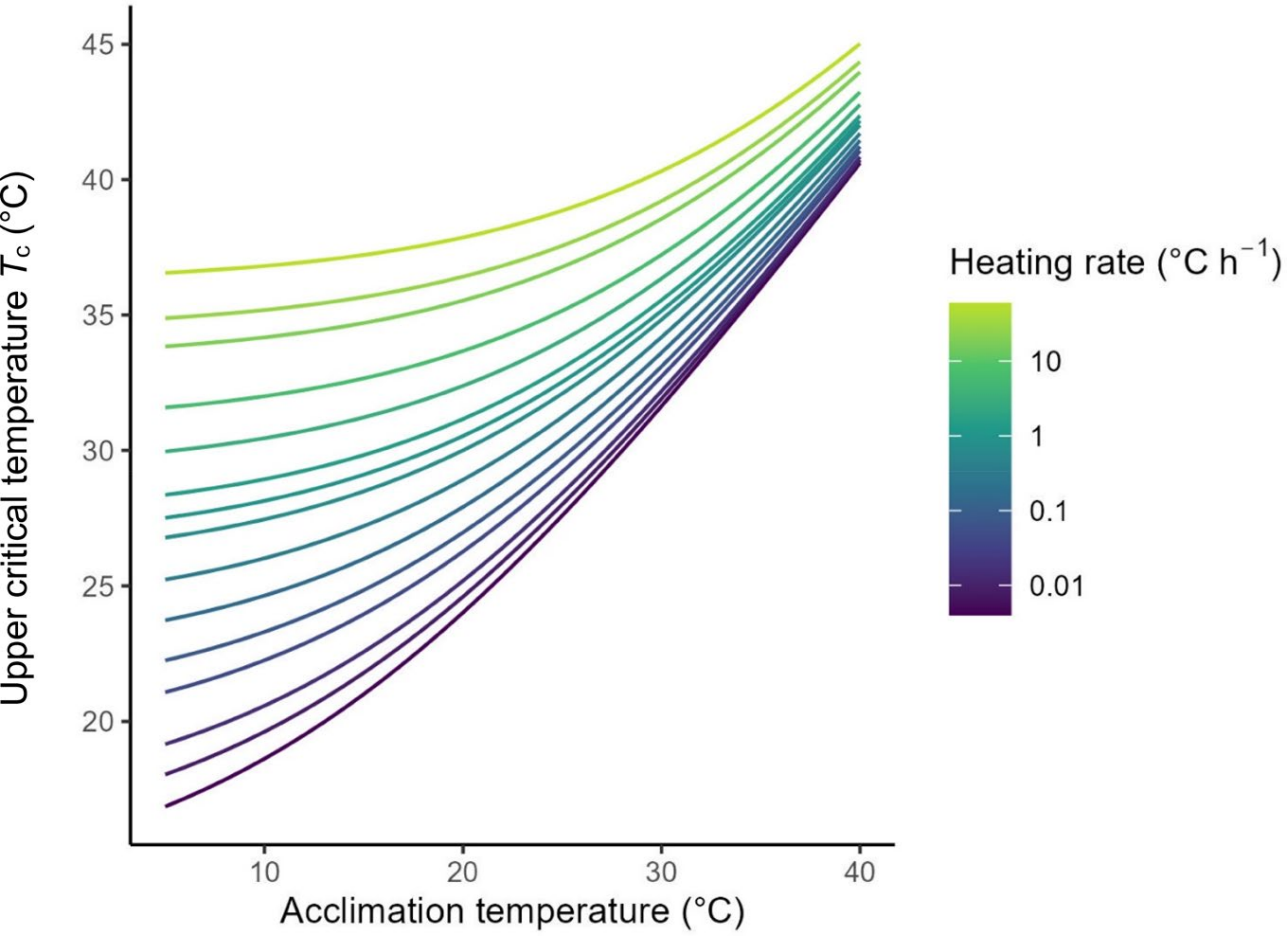
Upper critical temperature (*T*_c_) can be estimated for a range of acclimation temperatures (*T*_a_) and heating rates (colours) by measuring one *T*_c_ for one *T*_a_ and heating rate, and holding rescaled thermal tolerance as fixed (as the data suggest is the case). Median values of heating rate and *T*_c_ at a lower or higher acclimation temperature, as well as a value of 0.76 for γ from the data were used to calculate upper critical temperature (see Supplementary Section 6).

Because rescaled heating tolerance appears so well conserved within a species (i.e. no data fall very far from the identity lines in Figure 3b), and heating rate has a reasonably repeatable influence on that metric (Figure S4B), we can create generalised predictions of how an organism's thermal tolerance limit (*T*_c_) will vary when it is acclimated to any temperature and then heated at any rate; an example of such a prediction is shown for a hypothetical organism in Figure 5, where we used the median values of *T*_a_, *T*_c_ and heating rate across our full dataset (see Supplementary Material for the computational steps).

## 4 DISCUSSION

It is well known that survival probability is determined by the magnitude and duration of thermal stress. Nonlinear relationships between temperature and survival time were described more than a century ago (Bigelow, 1921) and are now incorporated into concepts of thermal tolerance ‘landscapes’ (Rezende et al., 2014). So strong is the temperature–time interaction that quantifying it for a species can subsequently allow accurate predictions of survival times at other static high temperatures, or critical temperatures under different dynamic heating rates (Jørgensen et al., 2019, 2021; Kingsolver & Umbanhowar, 2018; Rezende et al., 2014; Santos et al., 2011). Implicit in this earlier work (and sometimes explicit; Santos et al., 2011) is that thermal limits of an organism have a dependency with the scaling of biological rates with temperature; in part because there exists a temperature–time interaction at all, and in part because of the exponential shape of temperature‐survival curves. We take this work further by directly invoking the temperature dependence of metabolism and find that doing so very precisely explains the magnitude by which thermal limits shift when ectothermic animals are acclimated to different temperatures. In doing so, our study helps reconcile the fields of physiological thermal tolerance and the Metabolic Theory of Ecology (MTE).

Empirically speaking, the potential usefulness of our approach can be appreciated when we note the improvement that rescaling provides for predicting a species' heating tolerance based on measured heating tolerance at one other temperature (e.g. Figure 3a vs. 3b and Figure 4). The *R* ^2^ for predicted versus observed heating tolerance goes from ~52% to 94% (the stepwise improvement in accuracy and precision can be seen in the Supplementary Material for less to more derived versions of our model). These fits are comparable to those furnished under thermal death time models, but a key difference is that our approach requires no measurements of how thermal limits scale with temperature—we assume the scaling a priori based mathematically only on MTE. This represents an important advance, because it is not necessary to measure *T*_c_ at a variety of different *T*_a_ in order to understand how failure scales with temperature (the kind of multiple measurements required with the commonly used ‘*z* score’ approach); measuring *T*_c_ from just one starting temperature allows prediction of *T*_c_ from any other starting temperature with remarkable accuracy simply by accounting for the single curve that describes the average universal scaling of metabolic rate with temperature (Brown et al., 2004; Figure 4). Because the rescaled heating tolerance of a species appears so fixed and the effect of heating rate so repeatable, it is possible to predict what a species' *T*_c_ will be when heated from any starting temperature at any heating rate, as long as *T*_c_ is measured from just one start temperature *T*_a_; an example is shown in Figure 5. Such an approach could be a powerful new means of predicting the relative resilience of species to new thermal regimes such as heat waves of various magnitudes and heating rates and requires very few prior measurements. Figure 5 also illustrates visually how the exponential scaling of metabolism with temperature (coupled with our discovery that Δ*t*_r_ is ~ constant within a species) creates a tendency for *T*_c_ to increase only marginally with increasing *T*_a_; a pattern observed for decades (Araujo et al., 2013; Gunderson & Stillman, 2015; Rohr et al., 2018; Sunday et al., 2011) and seen clearly in our raw data (Figure 2).

Conceptually speaking, perhaps the most consequential finding of our study is that organisms heated at a given rate have practically the same heating tolerance regardless of the temperature they are acclimated to, once heating tolerance is rescaled to account for the temperature dependence of biological rates. On the one hand, we might interpret this result as ectotherms completely re‐adjusting their heating tolerance when they are acclimated to a higher temperature. However, another perspective could be that organisms do not adjust their heating tolerance at all; instead it is a rigidly fixed property of a species that is unchanged regardless of the temperature they are acclimated to. Either way, our results build on other studies and provide a strong quantitative explanation for why ectotherm thermal tolerance limits vary in the observed manner across macroecological scales, including why they appear to increase only marginally with latitude (Araujo et al., 2013; Sunday et al., 2011) and in acclimation experiments (Gunderson & Stillman, 2015; Morley et al., 2019). We contend that those observed patterns do not reflect hard physiological boundaries restricting the evolution of heat limits (Araujo et al., 2013); they simply reflect a link between thermal tolerance and biological rates, which increase exponentially with temperature (as proposed earlier; Payne & Smith, 2017). This represents a conceptual shift in how we think about thermal tolerance limits, so we encourage other studies to adopt this perspective and invoke metabolic scaling to explore other aspects of thermal tolerance in ectotherms, especially future resilience to climate warming in nature.

The ecological relevance of laboratory‐derived tolerance data is regularly questioned, especially for rapid, acute heating experiments (Barnes et al., 2010; Clark et al., 2013; Norin et al., 2014; Payne et al., 2016), because the influence of temperature on organism performance operates via different processes across biological scales (Iverson et al., 2020; Rezende & Bozinovic, 2019; Sinclair et al., 2016). The striking consistencies in the maintenance of rescaled heating tolerances across diverse species, broad acclimation periods, temperatures and heating rates suggest the mechanisms underpinning our data are likely relevant to ectotherms undergoing heating events in nature. Indeed, there are an increasing number of studies showing correlations between acute organismal responses in the laboratory and biogeographical patterns (Deutsch et al., 2020; Payne et al., 2016, 2018, 2021; von Schmalensee et al., 2021). Accordingly, our study could complement other recent work that explores the influence of different types of heating events, including fluctuating temperatures (Jørgensen et al., 2021), with a view to understand the resilience of organisms exposed to natural heating events in the wild (Bertolini & Pastres, 2021).

It is interesting that a single Arrhenius curve does such a good job of rescaling heat limits given organism ‘performance’ is generally described by thermal performance curves that include an optimum and then a rapid decline towards higher temperatures. Thermal tolerance limits are often considered disconnected from the scaling of biological rates with temperature, with the former reflecting structural phenomena (e.g. membrane fluidity; Bowler, 2018), and the latter reaction kinetics (Brown et al., 2004). However, structural and reaction rate processes being different mechanisms do not necessarily mean the two are not linked. For example, Bowler (2018) proposed that increases in membrane fluidity are likely to be the ultimate cause of organism death at high temperatures, with thermal perturbation of the plasma membrane precipitating a failure of ion pumps and nutrient transport, impairment of mitochondrial function, a breakdown of homeostasis and, eventual, cell death. But between the ultimate cause of membrane fluidity and proximate causes of cell death, a cascade of processes occurs that are manifested as rates (e.g. rate of nutrient transport or leakage of ions), so it could be expected that the onset of whole‐organism failure is indeed regulated by the rate at which biological processes proceed, which are themselves temperature‐dependent.

We do not know what combination of specific rate processes regulates an ectotherm's tolerance limit, but they seem to be general mechanisms because our dataset consisted of a wide diversity of species from seven Phyla, and all of them conformed to having a relatively fixed rescaled heating tolerance Δ*t*_r_ at different acclimation temperatures (Figure 2b). That said, while Δ*t*_r_ appears to be an approximately fixed property of a species (at a given heating rate), it remains highly variable among species even after heating rate is accounted for (Figure 3). Some of this variation may come from the different experimental protocols and thermal limit metrics used for different taxa in different experiments. But even more variation could arise from species differences in the total cumulative departure from homeostasis tolerated by various species before particular cells, tissues and organisms no longer function (if that departure is indeed what rescaled heating tolerance represents), and adaptation undoubtedly plays a role too. While rescaled heating tolerance appears remarkably fixed within a species, it also varies considerably with heating rate (following a negative ¾ power dependency, with faster heating reducing Δ*t*_r_ but increasing *T*_c_: the average effect of heating rate on *T*_c_ can be seen in Figure 5), and the effect of heating rate varied somewhat between species adding another source of unexplained variation. The negative influence of heating rate on rescaled heating tolerance was unexpected, but could reflect some mismatch between supply and demand that becomes increasingly severe as the biological system changes at a faster rate. Few studies have explored how heating rate influences energy turnover, but a recent study showed the aerobic metabolic rate of shrimp increases as they are heated at faster rates (Harding et al., 2023), possibly suggesting faster heating provides less time to downregulate metabolism at higher temperatures (as occurs via acclimation over longer time scales; Schulte et al., 2011; Seebacher et al., 2015). Taken together, while our results show remarkably consistent rescaled heating tolerance for a particular species and heating rate, there is significant unexplained variation in Δ*t*_r_ across species, including in how heat tolerance responds to heating rate in different species. Further work could explore what underlies these interspecific differences and also identify the precise mechanistic links between metabolic scaling and thermal limits, because our data suggest those links are strong.

We do not contend to have identified any mechanistic (physiological) insight beyond the fact that the scaling of biological rates with temperature almost perfectly explains how an organism varies its physiological temperature limit when acclimated to different temperatures (as long as heating rate is the same). With the role of metabolic scaling in setting thermal tolerance now identified, a fruitful body of future work could seek to explain which species traits (body size, aquatic vs. terrestrial, etc.) cause variation in heating tolerance, including how they respond differently to variable heating rates. While many studies explore how temperature influences metabolic rates and many others study the thermal tolerance of ectotherms, few studies invoke both themes simultaneously. Our study provides compelling evidence that metabolic rates govern variation in thermal tolerance limits, so we encourage future research on this metabolism–tolerance intersection to better predict potential winners and losers under future climate change.

## AUTHOR CONTRIBUTIONS

Nicholas L. Payne, Andrew L. Jackson and Jean‐Francois Arnoldi conceptualised the study. Jean‐Francois Arnoldi, Andrew L. Jackson, Jacinta D. Kong, Nicholas L. Payne and James A. Smith developed the methodology. All authors conducted the investigation. Nicholas L. Payne acquired funding. Nicholas L. Payne, Andrew L. Jackson, Jacinta D. Kong and Jean‐Francois Arnoldi administered the study. Nicholas L. Payne, Jean‐Francois Arnoldi and Jacinta D. Kong drafted the manuscript, and all other authors contributed substantially to revisions.

## CONFLICT OF INTEREST STATEMENT

The authors declare no competing interests.

## Supporting information


**Supplementary File S1.** Supplementary methods, results, and code used in the study.

**Supplementary File S2.** Expanded mathematical explanation of our derivation of the exponential approximation of the Arrhenius form.

## Data Availability

The code and the data are available at https://github.com/jacintak/rescaled_heating_tolerance. Data are available from the Zenodo Digital Repository https://doi.org/10.5281/zenodo.7387927 (Payne et al., 2025).

## References

[jane70042-bib-0001] Araujo, M. B. , Ferri‐Yanez, F. , Bozinovic, F. , Marquet, P. A. , Valladares, F. , & Chown, S. L. (2013). Heat freezes niche evolution. Ecology Letters, 16, 1206–1219. 23869696 10.1111/ele.12155

[jane70042-bib-0002] Barnes, D. K. A. , Peck, L. S. , & Morley, S. A. (2010). Ecological relevance of laboratory determined temperature limits: Colonization potential, biogeography and resilience of Antarctic invertebrates to environmental change. Global Change Biology, 16, 3164–3169.

[jane70042-bib-0003] Bertolini, C. , & Pastres, R. (2021). Tolerance landscapes can be used to predict species‐specific responses to climate change beyond the marine heatwave concept: Using tolerance landscape models for an ecologically meaningful classification of extreme climate events. Estuarine, Coastal and Shelf Science, 252, 107284.

[jane70042-bib-0004] Bigelow, W. (1921). The logarithmic nature of thermal death time curves. The Journal of Infectious Diseases, 29, 528–536.

[jane70042-bib-0005] Bowler, K. (2018). Heat death in poikilotherms: Is there a common cause? Journal of Thermal Biology, 76, 77–79. 30143300 10.1016/j.jtherbio.2018.06.007

[jane70042-bib-0006] Brown, J. H. , Gillooly, J. F. , Allen, A. P. , Savage, V. M. , & West, G. B. (2004). Toward a metabolic theory of ecology. Ecology, 85, 1771–1789.

[jane70042-bib-0007] Clark, T. D. , Sandblom, E. , & Jutfelt, F. (2013). Aerobic scope measurements of fishes in an era of climate change: Respirometry, relevance and recommendations. Journal of Experimental Biology, 216, 2771–2782. 23842625 10.1242/jeb.084251

[jane70042-bib-0008] Cook, A. M. , Rezende, E. L. , Petrou, K. , & Leigh, A. (2024). Beyond a single temperature threshold: Applying a cumulative thermal stress framework to plant heat tolerance. Ecology Letters, 27(3), e14416. 38549256 10.1111/ele.14416

[jane70042-bib-0009] Dell, A. I. , Pawar, S. , & Savage, V. M. (2011). Systematic variation in the temperature dependence of physiological and ecological traits. Proceedings of the National Academy of Sciences of the United States of America, 108, 10591–10596. 21606358 10.1073/pnas.1015178108 PMC3127911

[jane70042-bib-0010] Deutsch, C. , Penn, J. L. , & Seibel, B. (2020). Metabolic trait diversity shapes marine biogeography. Nature, 585, 557–562. 32939093 10.1038/s41586-020-2721-y

[jane70042-bib-0011] Einum, S. , & Burton, T. (2023). Divergence in rates of phenotypic plasticity among ectotherms. Ecology Letters, 26(1), 147–156. 36450612 10.1111/ele.14147 PMC10099672

[jane70042-bib-0012] Gunderson, A. R. , Dillon, M. E. , & Stillman, J. H. (2017). Estimating the benefits of plasticity in ectotherm heat tolerance under natural thermal variability. Functional Ecology, 31, 1529–1539.

[jane70042-bib-0013] Gunderson, A. R. , & Stillman, J. H. (2015). Plasticity in thermal tolerance has limited potential to buffer ectotherms from global warming. Proceedings of the Biological Sciences, 282, 20150401. 25994676 10.1098/rspb.2015.0401 PMC4455808

[jane70042-bib-0014] Harding, L. , Jackson, A. L. , & Payne, N. (2023). Energetic costs increase with faster heating in an aquatic ectotherm. Conservation Physiology, 11, coad042. 38026795 10.1093/conphys/coad042 PMC10660381

[jane70042-bib-0015] Hoffmann, A. A. , Chown, S. L. , & Clusella‐Trullas, S. (2013). Upper thermal limits in terrestrial ectotherms: How constrained are they? Functional Ecology, 27, 934–949.

[jane70042-bib-0016] Iverson, E. N. K. , Nix, R. , Abebe, A. , & Havird, J. C. (2020). Thermal responses differ across levels of biological organization. Integrative and Comparative Biology, 60, 361–374. 32483618 10.1093/icb/icaa052

[jane70042-bib-0017] Jørgensen, L. B. , Malte, H. , Ørsted, M. , Klahn, N. A. , & Overgaard, J. (2021). A unifying model to estimate thermal tolerance limits in ectotherms across static, dynamic and fluctuating exposures to thermal stress. Scientific Reports, 11, 12840. 34145337 10.1038/s41598-021-92004-6 PMC8213714

[jane70042-bib-0018] Jørgensen, L. B. , Malte, H. , & Overgaard, J. (2019). How to assess Drosophila heat tolerance: Unifying static and dynamic tolerance assays to predict heat distribution limits. Functional Ecology, 33, 629–642.

[jane70042-bib-0019] Kingsolver, J. G. , & Umbanhowar, J. (2018). The analysis and interpretation of critical temperatures. The Journal of Experimental Biology, 221, jeb167858. 29724777 10.1242/jeb.167858

[jane70042-bib-0020] Molina, A. N. , Carter, M. J. , & Rezende, E. L. (2024). Plasticity cannot fully compensate evolutionary differences in heat tolerance across fish species. Evolution, 78(12), 1949–1957. 39258466 10.1093/evolut/qpae126

[jane70042-bib-0021] Morley, S. A. , Bates, A. E. , Lamare, M. , Richard, J. , Nguyen, K. D. , Brown, J. , & Peck, L. S. (2016). Rates of warming and the global sensitivity of shallow water marine invertebrates to elevated temperature. Journal of the Marine Biological Association of the United Kingdom, 96, 159–165.

[jane70042-bib-0022] Morley, S. A. , Peck, L. S. , Sunday, J. , Heiser, S. , & Bates, A. E. (2018). Acclimation potential of global ectothermic species, collated from literature, 1960 to 2015. Natural Environment Research Council, Polar Data Centre.

[jane70042-bib-0023] Morley, S. A. , Peck, L. S. , Sunday, J. M. , Heiser, S. , & Bates, A. E. (2019). Physiological acclimation and persistence of ectothermic species under extreme heat events. Global Ecology and Biogeography, 28, 1018–1037.

[jane70042-bib-0024] Norin, T. , Malte, H. , & Clark, T. D. (2014). Aerobic scope does not predict the performance of a tropical eurythermal fish at elevated temperatures. Journal of Experimental Biology, 217, 244–251. 24115064 10.1242/jeb.089755

[jane70042-bib-0025] Payne, N. L. , Kong, J. D. , Jackson, A. L. , Bates, A. E. , Morley, S. A. , Smith, J. A. , & Arnoldi, J. F. (2025). Data from: Heat limits scale with metabolism in ecothermic animals. *Zenodo Digital Repository*. 10.5281/zenodo.7387927 PMC12134445 40356272

[jane70042-bib-0026] Payne, N. L. , Meyer, C. G. , Smith, J. A. , Houghton, J. D. R. , Barnett, A. , Holmes, B. J. , Nakamura, I. , Papastamatiou, Y. P. , Royer, M. A. , Coffey, D. M. , Anderson, J. M. , Hutchinson, M. R. , Sato, K. , & Halsey, L. G. (2018). Combining abundance and performance data reveals how temperature regulates coastal occurrences and activity of a roaming apex predator. Global Change Biology, 24, 1884–1893. 29516588 10.1111/gcb.14088

[jane70042-bib-0027] Payne, N. L. , Morley, S. A. , Halsey, L. G. , Smith, J. A. , Stuart‐Smith, R. , Waldock, C. , & Bates, A. E. (2021). Fish heating tolerance scales similarly across individual physiology and populations. Communications Biology, 4, 264. 33649450 10.1038/s42003-021-01773-3 PMC7921436

[jane70042-bib-0028] Payne, N. L. , & Smith, J. A. (2017). An alternative explanation for global trends in thermal tolerance. Ecology Letters, 20, 70–77. 27905195 10.1111/ele.12707

[jane70042-bib-0029] Payne, N. L. , Smith, J. A. , van der Meulen, D. E. , Taylor, M. D. , Watanabe, Y. Y. , Takahashi, A. , Marzullo, T. A. , Gray, C. A. , Cadiou, G. , & Suthers, I. M. (2016). Temperature dependence of fish performance in the wild: Links with species biogeography and physiological thermal tolerance. Functional Ecology, 30, 903–912.

[jane70042-bib-0030] Pottier, P. , Burke, S. , Zhang, R. Y. , Noble, D. W. , Schwanz, L. E. , Drobniak, S. M. , & Nakagawa, S. (2022). Developmental plasticity in thermal tolerance: Ontogenetic variation, persistence, and future directions. Ecology Letters, 25, 2245–2268. 36006770 10.1111/ele.14083 PMC9804923

[jane70042-bib-0031] Rezende, E. L. , & Bozinovic, F. (2019). Thermal performance across levels of biological organization. Philosophical Transactions of the Royal Society, B: Biological Sciences, 374, 20180549. 10.1098/rstb.2018.0549 PMC6606466 31203764

[jane70042-bib-0032] Rezende, E. L. , Bozinovic, F. , Szilágyi, A. , & Santos, M. (2020). Predicting temperature mortality and selection in natural Drosophila populations. Science, 369, 1242–1245. 32883867 10.1126/science.aba9287

[jane70042-bib-0033] Rezende, E. L. , & Carter, M. J. (2024). Cumulative heat stress in fluctuating temperatures and implications for the distribution of freshwater fish. Global Change Biology, 30(12), e17623. 39648972 10.1111/gcb.17623

[jane70042-bib-0034] Rezende, E. L. , Castañeda, L. E. , Santos, M. , & Fox, C. (2014). Tolerance landscapes in thermal ecology. Functional Ecology, 28, 799–809.

[jane70042-bib-0035] Rohr, J. R. , Civitello, D. J. , Cohen, J. M. , Roznik, E. A. , Sinervo, B. , & Dell, A. I. (2018). The complex drivers of thermal acclimation and breadth in ectotherms. Ecology Letters, 21, 1425–1439. 30009486 10.1111/ele.13107

[jane70042-bib-0036] Ruthsatz, K. , Dahlke, F. , Alter, K. , Wohlrab, S. , Eterovick, P. C. , Lyra, M. L. , Gippner, S. , Cooke, S. J. , & Peck, M. A. (2024). Acclimation capacity to global warming of amphibians and freshwater fishes: Drivers, patterns, and data limitations. Global Change Biology, 30, e17318. 38771091 10.1111/gcb.17318

[jane70042-bib-0037] Santos, M. , Castañeda, L. E. , & Rezende, E. L. (2011). Making sense of heat tolerance estimates in ectotherms: Lessons from Drosophila. Functional Ecology, 25, 1169–1180.

[jane70042-bib-0038] Schulte, P. M. , Healy, T. M. , & Fangue, N. A. (2011). Thermal performance curves, phenotypic plasticity, and the time scales of temperature exposure. Integrative and Comparative Biology, 51, 691–702. 21841184 10.1093/icb/icr097

[jane70042-bib-0039] Seebacher, F. , White, C. R. , & Franklin, C. E. (2015). Physiological plasticity increases resilience of ectothermic animals to climate change. Nature Climate Change, 5, 61–66.

[jane70042-bib-0040] Sinclair, B. J. , Marshall, K. E. , Sewell, M. A. , Levesque, D. L. , Willett, C. S. , Slotsbo, S. , Dong, Y. , Harley, C. D. , Marshall, D. J. , Helmuth, B. S. , & Huey, R. B. (2016). Can we predict ectotherm responses to climate change using thermal performance curves and body temperatures? Ecology Letters, 19, 1372–1385. 27667778 10.1111/ele.12686

[jane70042-bib-0041] Sunday, J. M. , Bates, A. E. , & Dulvy, N. K. (2011). Global analysis of thermal tolerance and latitude in ectotherms. Proceedings of the Royal Society B: Biological Sciences, 278, 1823–1830. 10.1098/rspb.2010.1295 PMC3097822 21106582

[jane70042-bib-0042] Terblanche, J. S. , Deere, J. A. , Clusella‐Trullas, S. , Janion, C. , & Chown, S. L. (2007). Critical thermal limits depend on methodological context. Proceedings of the Royal Society B: Biological Sciences, 274, 2935–2943. 10.1098/rspb.2007.0985 PMC2291155 17878142

[jane70042-bib-0043] von Schmalensee, L. , Hulda Gunnarsdóttir, K. , Näslund, J. , Gotthard, K. , & Lehmann, P. (2021). Thermal performance under constant temperatures can accurately predict insect development times across naturally variable microclimates. Ecology Letters, 24(8), 1633–1645. 34036719 10.1111/ele.13779

