# Supplementary material for: Heat limits scale with metabolism in ectothermic animals
Source: J Anim Ecol. 2025 May 12;94(6):1307–16. doi: 10.1111/1365-2656.70042 (PMC12134445; doi:10.1111/1365-2656.70042)
Supplement: Supplementary file 1 — Supplementary File S1. Supplementary methods, results, and code used in the study. [file JANE-94-1307-s001.HTM]

Heat limits scale with metabolism in ectothermic animals


## Table of contents

- 1 Heating tolerance data
  - 1.1 Figure 2A
- 2 Rescaling heating tolerance via heating duration
  - 2.1 Data requirements
  - 2.2 Describing non-linear relationship between biological rates and temperature
  - 2.3 Defining functions and notation
  - 2.4 Code to rescale heating tolerances
  - 2.5 Figure 2B
- 3 Sensitivity of activation energy
- 4 Predicting heating tolerance for a new acclimation temperature
  - 4.1 Assuming heating tolerances are the same
  - 4.2 Correcting for acclimation temperature difference
  - 4.3 Account for difference in acclimation temperature and change in temperature
  - 4.4 Figure 3
- 5 The effect of heating rate on rescaled heating duration
  - 5.1 Testing a null effect of heating rates on rescaled heating duration
  - 5.2 Figure 4A
  - 5.3 Figure 4B
  - 5.4 Quantifying the effect of heating rates on rescaled heating duration
  - 5.5 Figure 4C
  - 5.6 Interspecific scaling exponent of \(\gamma\)
- 6 Generalised graphs of rescaled heating durations
  - 6.1 Figure 5
- 7 Table of equations
- 8 Manuscript Figures
- 9 Session information
- 10 References

# Heat limits scale with metabolism in ectothermic animals

 Code

- Show All Code
- Hide All Code
- ---
- View Source

Supplementary material

Authors

Affiliation

Jacinta Kong

Trinity College Dublin, Ireland

Andrew L Jackson

Trinity College Dublin, Ireland

Nicholas Payne

Trinity College Dublin, Ireland

Jean François Arnoldi

Trinity College Dublin, Ireland

Published

February 24, 2025

**README**  
This document presents the full workflow and code of *Heating tolerance of ectotherms scales with temperature’s non-linear influence on biological rates*, including data processing and generating figures for the study. The analysis was conducted in R version 4.4.2 (2024-10-31) (R Core Team 2022) running `Tidyverse` (Wickham 2019) & `cowplot` (Wilke 2020) (Section 9). Supplementary analysis and notation are presented here. See Methods in main text for the primary mathematical notation and derivation, and Table S 3 for the order of equations.

---

# 1 Heating tolerance data

Code

```
# Import Morley et al 2018
ARR <- readxl::read_excel("data/Morley_et_al_(2018)_-_Collated_Species_Acclimation_Data.xlsx",  skip = 8) %>% 
  # Add unique id
  rowid_to_column("row_ID") %>%  
  # remove blank rows
  filter(!is.na(Taxon)) %>%
  # rename columns
  rename("ta_high" = 11, #"High acclimation Temperature /°C" ,
         "ta_low" = 12, #"Low Acclimation Temperature/ °C" ,
         "ARR" = 17, #"CTMax ARR" ,
         "tc_high" = 16, #"CTMax/ °C", # CT max of the high acclimation temperature
         "acclim_window" = 13, #"Acclimation Window /°C",
         "heating_rate" = 22, #"°C change hr-1",
         "collection_latitude" = 19) %>%  #"Latitude of Collection/ °") %>%
  # Fix typo in taxon
  mutate(Taxon = str_replace_all(Taxon, "amphibains", "amphibians")) %>%
  # Fix incorrect heating rate in DOI 10.1007/s10695-013-9793-7
  mutate(heating_rate = ifelse(row_ID  == 367, (0.3 * 60), heating_rate)) %>%
  # Calculate low temperature CT max
  mutate(tc_low = tc_high - (ARR * acclim_window)) 


# Calculate heating tolerances for each acclimation temperature, H (Equation 1)
ARR <- ARR %>% 
  mutate(ht_low = tc_low - ta_low,
         ht_high = tc_high - ta_high)
```

We reanalyzed a dataset on thermal tolerances of diverse ectotherms containing Acclimation Response Ratios (ARR), acclimation temperatures (\(T\_a\)) and upper thermal tolerance limits (\(T\_c\)) compiled from the literature between 1960 – 2015 (Morley et al. 2018, 2019). The original dataset is available from the Polar Data Centre under an Open Government Licence v3.0 (Morley et al. 2018). Specifically the file is named “Morley et al (2018) - Collated Species Acclimation Data.xlsx” on the repository but the downloaded file has white space replaced with “\_“. Ectotherms were acclimated for between 1 and 300 days to a lower temperature or a higher temperature for a paired observation for each species.

Data were only included if each species had thermal tolerances measured for two different starting acclimation temperatures with an average of 10 °C apart, allowing us to compare heating tolerances both within and among species. The dataset contained 369 observations of 316 species from 15 Classes from 7 Phyla: Chordata, Arthropoda, Mollusca, Platyhelminthes, Echinodermata, Brachiopoda and Cnidaria). These species spanned northern and southern hemispheres, polar to tropical latitudes, and freshwater, marine and terrestrial habitats.

Animals were heated under a constant ramping temperature from their acclimation temperature until the point of organismal collapse, which we refer to here as their upper thermal tolerance limit (\(T\_c\)), called CTmax in Morley et al. (2019). A small proportion of observations used lethal temperatures as the endpoint and we did not exclude these observations but do not consider our analysis to appropriately apply to mortality rates or liklihood of survival. Within paired observations, each species was measured at the same constant heating rate, and heating rates varied among paired observations representing independent species or experimental assays. Thermal tolerance limits were assayed for both acclimation temperatures in paired observations for a species (referred as `tc_low` or `tc_high` in this code).

The upper thermal tolerance limit of the lower acclimation temperature was not reported in Morley et al. (2019). We derived the upper thermal tolerance limit of the lower acclimation temperature from rearranging the formula for Acclimation Response Ratios. We first multiplied the acclimation response ratio for each species by the difference in acclimation temperatures; called acclimation window (`acclim_window`) following Morley et al. (2019). We then subtracted this result from the upper thermal tolerance of the higher acclimation temperature (`tc_high`) for each species to calculate the upper thermal tolerance of the lower acclimation temperature (`tc_low`).

\[
T\_{c \space lower} = T\_{c \space higher} - (ARR \times acclimation \space window)
\]

## 1.1 Figure 2A

Generate Figure 2A of heating tolerance.

Code

```
heating_graph <- ARR %>% 
  ggplot(aes(ht_low, ht_high, col = heating_rate)) +
  geom_abline(slope = 1, intercept = 0, lty = 2, alpha = 0.6) +
  geom_smooth(method = "lm", se = FALSE, col = 1) + 
  geom_point() +
  labs(x = expression("Lower acclimation temperature H (\u00B0C)"),
       y = expression("Higher acclimation temperature H (\u00B0C)"),
       title = expression("Heating tolerance, H")) +
  scale_x_continuous(limits = c(3,40), expand = c(0,0), trans = "log10") +
  scale_y_continuous(limits = c(3,40), expand = c(0,0), trans = "log10") +
  coord_equal() +  
  scale_colour_viridis_c(name = expression("Heating rate (\u00B0C h" ^-1 * ")"), end = 0.9, trans = "log10", labels = function(x) signif(x))

heating_graph
```

Heating tolerances at the lower and higher acclimation temperatures for paired observations on Log 10 transformed axes. Figure 2A in main text.

---

# 2 Rescaling heating tolerance via heating duration

## 2.1 Data requirements

Rescaling heating tolerances requires an assay of thermal limits using a ramping protocol with a constant heating rate. The required variables are upper thermal tolerance limit (\(T\_c\)), acclimation temperature or the starting temperature of the ramping assay (\(T\_a\)), and heating rate of the ramping assay (heating rate). These variables are used to calculate heating tolerance and elapsed duration (heating duration) of the ramping assay. Acclimation and starting temperature are used interchangeably as acclimation temperature is the starting temperature of the heating assay in our dataset. Table S 1 contains a summary of the main mathematical notation used.

Our calculations can be generalised to any heating event with a constant heating rate or a known duration of time at a constant temperature. Our model does not formally apply to other protocols of assaying thermal tolerance limits or to lower thermal tolerance limits but these variables present avenues for future studies. Rescaling heating duration and heating tolerance is dependent on three assumptions: 1) Heating tolerance is arbitrarily defined with respect to 0 °C, 2) heating rate is constant, and 3) the Universal Temperature Dependence (Boltzmann-Arrhenius equation) is not affected by the physiological state of the organism or the experimental conditions (i.e., activation energy (\(E\)) does not change).

## 2.2 Describing non-linear relationship between biological rates and temperature

Heating tolerances represent a duration of time (heating duration, \(\Delta t\)) between two time points (\(t\_a\), the start of the heating assay, and \(t\_c\), the end of the heating assay) that have a corresponding temperature (acclimation temperature, \(T\_a\) and upper thermal tolernace \(T\_c\), respectively).

Therefore, as we can expect biological processes occurring during heating to scale with temperature we can also expect the durations of theses biological processes at one temperature, \(\Delta t(T)\), to scale with temperature. We can describe the relationship between heating durations starting from two temperatures (\(T\_{a1}\) and \(T\_{a2}\)) as:

\[
\frac{\Delta t(T\_{a1})}{\Delta t(T\_{a2})} \approx e^{\frac{E}{kT\_{a1}}-\frac{E}{kT\_{a2}}}
\tag{1}\]

In Equation S 1, \(\Delta t\) are elapsed durations of the heating assay starting at the acclimation temperature, \(E\) is the activation energy of the Universal Temperature Dependence (0.66 eV) and \(k\) is Boltzmann’s constant. Following Equation S 1, we can use a reference temperature to normalize heating durations to a normalized temperature scale (\(\tau\)). We use 0 °C as the reference temperature and define a normalization constant (\(T\_K\)) with a value of 273.15 (i.e. 0°C in Kelvin) for centering temperatures (\(T\)) on the Celsius scale.

\[
\tau = \frac{T}{T\_K}
\] Here, \(T\) are temperatures on the Celsius scale and \(\tau\) are normalised temperatures that are centred around the normalisation constant \(T\_K\). Normalised temperature \(T\_K\) is unitless and can be converted back into degree Celsius by \(T=\tau \times T\_K\).

Code

```
# Constants in Equation S1
# Boltzmann's constant, k, in units of eV K-1
k <- 8.61733 * 10 ^ -5
# Normalisation constant to center around 273.15K (T_K)
Tk <- 273.15
```

Substituting the normalisation constant \(T\_K\) as \(T\_{a1}\) and temperature \(T\) (in °C) as \(T\_{a2}\) in Equation S 1, we can derive an initial expression that rescales heating durations by accounting for acclimation temperature:

\[\Delta t\_r = e^{\frac{E}{kT\_K}}e^{-\frac{E}{kT}} \Delta t(T)\] Subscript \(r\) denotes variables rescaled by the non-linear temperature dependence of biological rates (Table S 1). \(\Delta t\_r\) are rescaled heating durations that correspond to non-rescaled heating duration (\(\Delta t\)).

Rescaled heating duration can be written as an integral between the start (\(t\_a\)) and end times (\(t\_c\)) of the heating assay, assuming a constant temperature ramp:

\[
\Delta t\_r = e^{\frac{E}{kT\_K}} \int\_{t\_a}^{t\_c} e^{-\frac{E}{kT(t)}} dt
\tag{2}\]

We can simplify Equation S 2 by using the normalised temperature scale (\(\tau\)) which removes the dimension of temperature. On a normalised temperature scale (\(\tau\)), \(\frac{E}{kT}\) in Equation S 2 becomes:

\[
\frac{E}{kT} = {\frac{E}{kT\_K}} {\frac{1}{1 + \frac{T}{T\_K}}} \\
= {\frac{E}{kT\_K}} {\frac{1}{1 + \tau}} \\
\approx \beta (1-\tau)
\]

Where

\[
\beta = \frac{E}{k T\_K}
\tag{3}\]

Code

```
# define \beta for a given activation energy, E = 0.66 (Equation S3)
beta_K <- 0.66 / (k * Tk)
```

\(\beta\) is a constant constructed from activation energy \(E\), Boltzmann’s constant \(k\), and the reference temperature \(T\_K\) with an approximate value of 28 using \(E =\) 0.66 (`beta_K`) (Equation S 3). Following Equation 3 in the main text, we can use Equation S 3 to approximate the non-linear thermal dependence of biological rates and derive Equations 4 and 5 in the main text for calculating rescaled heating durations accounting for heating rate and non-linear rate-time relationships.

## 2.3 Defining functions and notation

Define functions from main text to rescale heating duration. Equation 6 uses both `delta_tr_fun` and `g_beta_fun`.

Code

```
# function g of x and the constant beta (Equation 6)
g_beta_fun <- function(x){(exp(beta_K * x) - 1) / beta_K}

# function for delta t_r that uses g_beta_fun (Equation 6)
delta_tr_fun <- function(tau_a, tau_c, lambda){
  exp(beta_K * tau_a) / lambda * g_beta_fun(tau_c - tau_a)
}

# function f of x and the constant beta (Equation 7)
f_beta_fun <- function(x){log(1 + beta_K * x) / beta_K}
```

Table 1: Main mathematical notation and description used in this analysis.

| Symbol | Description |
| --- | --- |
| \(H\) | Heating tolerance; range of temperatures between acclimation temperature (\(T\_a\)) and upper thermal tolerance (\(T\_c\)) |
| \(\Delta t\) | Elapsed duration of a heating assay between the start (\(t\_a\)) and end (\(t\_c\)) time points |
| \(\_r\) | Variables rescaled by the temperature-dependence of biological rates |
| \(\_a\) | Variables referring to acclimation or starting temperature of a heating assay |
| \(\_c\) | Variables referring to the upper thermal tolerance limit of a heating assay |
| \(\tau\) | Normalised temperature scale centred around \(T\_K\) (normalisation constant 273.15K) |
| \(\lambda\) | Normalised heating rate centred around \(T\_K\) |
| \(\gamma\) | Scaling exponent for the effect of heating rate \(\lambda\) on rescaled heating duration \(\Delta t\_r\) |

## 2.4 Code to rescale heating tolerances

Code

```
# Step 1. Normalise acclimation temperature, upper thermal tolerances and heating rate by the normalisation constant (Tk, called tau_*), then calculate normalised heating tolerance (called delta_tau_*)
ARR <- ARR %>% 
  mutate(
    # acclimation temperatures
    tau_a_low = ta_low / Tk,
    tau_a_high = ta_high / Tk,
    # upper thermal tolerances
    tau_c_low = tc_low / Tk,
    tau_c_high = tc_high / Tk,
    # heating rate
    lambda = heating_rate / Tk,
    # normalised heating tolerance
    delta_tau_low = tau_c_low - tau_a_low,
    delta_tau_high = tau_c_high - tau_a_high)

# Step 2. Calculate rescaled heating duration for lower acclimation temperatures using Equation 6
ARR <- ARR %>% 
  mutate(delta_tr_low = pmap_dbl(list(tau_a = tau_a_low,
                                  tau_c = tau_c_low,
                                  lambda = lambda),
                             delta_tr_fun))

# Step 3. Calculate rescaled heating duration for higher acclimation temperatures using Equation 6
ARR <- ARR %>% 
  mutate(delta_tr_high = pmap_dbl(list(tau_a = tau_a_high,
                                   tau_c = tau_c_high,
                                   lambda = lambda),
                              delta_tr_fun))
```

## 2.5 Figure 2B

Generate Figure 2B in the main text of rescaled heating duration.

Code

```
rescaled_duration <- ggplot(data = ARR,
                            mapping = aes(x = delta_tr_low,
                                          y = delta_tr_high,
                                          col = heating_rate)) +
  geom_abline(slope = 1, intercept = 0, lty = 2, alpha = 0.6) +
  geom_smooth(method = "lm", se = FALSE, col = 1) +
  geom_point() +
  scale_x_continuous(trans = "log10", labels = function(x) round(x), expand = c(0,0), limits = c(0.1, 10000)) +
  scale_y_continuous(trans = "log10", labels = function(x) round(x), expand = c(0,0), limits = c(0.1, 10000)) +
  coord_equal() +
  labs(x = expression("Lower acclimation temperature " * Delta * t[r]),
       y = expression("Higher acclimation temperature " * Delta * t[r])) +
  ggtitle(expression(paste("Rescaled heating duration, ") * Delta * t[r])) +
  scale_colour_viridis_c(name = expression("Heating rate (\u00B0C h" ^-1 * ")"), end = 0.9, trans = "log10", labels = function(x) signif(x))

rescaled_duration
```

Rescaled heating duration at the lower and higher acclimation temperature on Log 10 axes have a strong dependency with heating rate (colours). Figure 2B in main text.

---

# 3 Sensitivity of activation energy

Interspecific activation energy of various thermally-dependent traits ranges between 0.2 and 1.2 with a median of 0.55 eV and mean of 0.66eV (Dell, Pawar, and Savage 2011). We investigated the sensitivity of our results to variation in activation energy across the range of activation energies reported in Dell, Pawar, and Savage (2011). `delta_tr_gen` is a generalised function to rescale heating tolerance with \(E\) as a variable. \(\beta\) values are calculated for each value of \(E\). We found changing activation energy and thus \(\beta\) does not meaningfully change the reported patterns (Figure S 1) and \(R^2\) values are above 0.9 across the range of activation energies used (Table S 2).

Code

```
# Generalised function for rescaling heating tolerance that accepts any activation energy
delta_tr_gen <- function(x, ev, k = 8.61733*10^-5, Tk = 273.15) { # ev is activation energy
  
  # calculate beta K constant (Equation S3)
  beta_K_sensi <- ev / (k * Tk)
  
  # function for delta tr that uses g_beta_fun (Equation 6)
  delta_tr_gen <- function(tau_a, tau_c, lambda){
    exp(beta_K_sensi * tau_a) / lambda * g_beta_fun_gen(tau_c - tau_a)
  }
  
  # function g of x and the constant beta (Equation 6)
  g_beta_fun_gen <- function(x){(exp(beta_K_sensi * x) - 1) / beta_K_sensi}
  
  # function f of x and the constant beta (Equation 7)
  f_beta_fun_gen <- function(x){log(1 + beta_K_sensi * x) / beta_K_sensi}
  
  # normalise Ta, Tc and heating rate by Tk
  x <- x %>% # Calculate heating rate as lambda
    mutate(tau_a_low = ta_low / Tk,
           tau_a_high = ta_high / Tk,
           tau_c_low = tc_low / Tk,
           tau_c_high = tc_high / Tk,
           lambda = heating_rate / Tk,
           delta_tau_low = tau_c_low - tau_a_low, # calculate delta taus
           delta_tau_high = tau_c_high - tau_a_high)

  # Rescale lower temperature experiments
  x <- x %>% 
    mutate(delta_tr_low = pmap_dbl(list(tau_a = tau_a_low,
                                    tau_c = tau_c_low,
                                    lambda = lambda),
                               delta_tr_gen))
  
  # Rescale higher temperature experiments
  x <- x %>% 
    mutate(delta_tr_high = pmap_dbl(list(tau_a = tau_a_high,
                                     tau_c = tau_c_high,
                                     lambda = lambda),
                                delta_tr_gen))
  
  # Add predictions of high from low and conversely low from high
  x <- x %>% 
    mutate(activation = ev,
           delta_tau_high_hat = f_beta_fun_gen(exp(beta_K_sensi * (tau_a_low - tau_a_high)) * g_beta_fun_gen(delta_tau_low)), # estimate high from low
           delta_tau_low_hat = f_beta_fun_gen(exp(beta_K_sensi * (tau_a_high - tau_a_low)) * g_beta_fun_gen(delta_tau_high))) # estimate low from high

  return(x) # as list
}
```

The dataset is replicated for a vector of activation energies, then the `delta_tr_gen` function is applied to each element of the list using `mapply`.

Code

```
# Sequence of activation energies between 0.2 and 1.2
activation <- seq(0.2, 1.2, 0.2)
# Repeat ARR dataset over length of activation energies
ARR_list <- replicate(length(activation), ARR, simplify = FALSE)
# Rescale heating tolerances, merge to single data frame
ARR_list <- do.call(rbind, mapply(ARR_list, FUN = delta_tr_gen, ev = activation, SIMPLIFY = FALSE))

# Create plot of results
sensi_plot <- ggplot(data = ARR_list, 
                 mapping = aes(x = delta_tr_low, 
                               y = delta_tr_high)) + 
  geom_point(col = "grey") + 
  geom_smooth(method = "lm", se = FALSE, col = "black") + 
  geom_abline(slope = 1, intercept = 0, lty = 2, alpha = 0.6) +
  scale_x_continuous(expand = c(0.01,0), trans = "log10", labels = function(x) format(x, scientific = FALSE)) +
  scale_y_continuous(expand = c(0.01,0), trans = "log10", labels = function(x) format(x, scientific = FALSE)) +
  facet_wrap(.~activation, ncol=3, scales = "free") +
  labs(x = expression("Lower temperature rescaled heating duration"),
       y = expression("Higher temperature rescaled heating duration")) +
  theme_classic(base_size = 12)

sensi_plot
```

Figure 1: rescaled heating durations for the lower and higher acclimation temperatures calculated using values of activation energy between 0.2 and 1.2 eV. Observations (points) with a fitted linear model (solid line) fall along the identity line (dashed line).

Code

```
ARR_list %>% 
  group_by(activation) %>% 
  group_map(~ summary(lm(delta_tr_high ~ delta_tr_low, data = .x))$r.squared) %>% 
  unlist() %>% 
  data.frame(activation = activation, "R2" = .) %>% 
  knitr::kable(digits = 3, col.names = c("Activation energy (eV)", "R squared value"))
```

Table 2: R2 values of the regression between rescaled heating durations for the lower and higher acclimation temperatures calculated using a range of activation energies.

| Activation energy (eV) | R squared value |
| --- | --- |
| 0.2 | 0.982 |
| 0.4 | 0.982 |
| 0.6 | 0.986 |
| 0.8 | 0.990 |
| 1.0 | 0.991 |
| 1.2 | 0.988 |

---

# 4 Predicting heating tolerance for a new acclimation temperature

To test the importance of considering acclimation temperature and the non-linear temperature-dependence of biological rates in our analysis, we investigated a series of hypotheses representing assumptions made in our analysis from the simplest model to the full derivation. We estimated the heating tolerance at the higher acclimation temperature (an unknown value, \(a2\)) from the heating tolerance of the lower acclimation temperature (a known value, \(a1\)), via rescaling. We compared our estimations against the observed upper thermal tolerances. If our estimations matched our observed values, then our formulations were suitable. If not, then our assumptions are not valid and further derivations are required.

## 4.1 Assuming heating tolerances are the same

The simplest formula to estimate a new heating tolerance from a new acclimation temperature is to assume that heating tolerances at the higher and lower acclimation temperature experiments are the same. For example, we estimated the heating tolerance at the higher acclimation temperature \(\Delta \tau (\tau\_{a2})\), from the lower acclimation temperature, \(\Delta \tau (\tau\_{a1})\).

\[
\Delta \tau (\tau\_{a2}) \approx \Delta \tau (\tau\_{a1})
\tag{4}\]

Code

```
pred1 <- ggplot(data = ARR,
       mapping = aes(y = delta_tau_low * Tk,
                     x = delta_tau_high * Tk)) + 
  geom_point() + 
  geom_abline(intercept = 0, slope = 1, lty = 2, alpha = 0.6) + 
  geom_smooth(method = "lm", se = FALSE, col = "darkgrey") + 
  labs(y = expression("Estimated heating tolerance (\u00B0C)"),
       x = expression("Observed heating tolerance (\u00B0C)")) + 
  scale_x_continuous(expand = c(0,0), limits = c(0, 40)) +
  scale_y_continuous(expand = c(0,0), limits = c(0, 40))

pred1
```

Figure 2: Observed vs estimated heating tolerance for the higher acclimation temperature (°C) assuming heating tolerance is the same as the lower acclimation temperature. Predictions were made for each species (points). Predictions should fall along a 1:1 identity line (dashed line) if assuming that the heating tolerance of the higher temperature is the same as at the lower temperature is accurate. The solid line is an ordinary least-squares regression.

Code

```
# high from low R2
pred_H0 <- lm(delta_tau_low ~  delta_tau_high, 
                   data = ARR) %>% 
  summary(.) %>%
  .$r.squared %>% 
  round(., 4) * 100
```

Assuming heating tolerance is the same at both acclimation temperatures overestimated the heating tolerance at the higher temperature (Equation S 4); points fall above the identity line (Figure S 2). The \(R^2\) is 51.9%.

## 4.2 Correcting for acclimation temperature difference

Next, we can account for the difference in acclimation temperatures by rescaling by the acclimation temperature via \(e^{\beta (\tau\_{a1} - \tau\_{a2})}\):

\[
\Delta \tau (\tau\_{a2}) \approx \Delta \tau (\tau\_{a1}) e^{\beta (\tau\_{a1} - \tau\_{a2})}
\tag{5}\]

Code

```
pred2 <- pred1 + aes(y = (delta_tau_low * exp(beta_K * (tau_a_low - tau_a_high))) * Tk) +
  labs(y = expression("Estimated heating tolerance (\u00B0C)")) 
pred2
```

Figure 3: Observed vs estimated heating tolerance for the higher acclimation temperature (°C) accounting for the change in acclimation temperature. Predictions were made for each species (points). Predictions should fall along a 1:1 identity line (dashed line) if the model is accurate. The solid line is an ordinary least-squares regression.

Code

```
# high from low R2
pred_H1 <- lm(((delta_tau_low * exp(beta_K * (tau_a_low - tau_a_high)))) ~  delta_tau_high, 
                   data = ARR) %>% 
  summary(.) %>%
  .$r.squared %>% 
  round(., 4) * 100
```

Accounting for the difference in acclimation temperature when rescaling heating tolerances underestimates thermal tolerances for the higher acclimation temperature (Equation S 5); points fall below the identity line (Figure S 3). The \(R^2\) is 76.73%.

## 4.3 Account for difference in acclimation temperature and change in temperature

Finally, we can derive a more precise formulation that fully accounts for the change to heating tolerance that occurs during the experiment as the temperature is increased using Equation 8 in the main text (below).

\[
\Delta \tau (\tau\_{a2}) \approx f\_\beta \big(e^{\beta (\tau\_{a1} - \tau\_{a2})} g\_\beta(\Delta \tau(\tau\_{a1})) \big)
\] To test the consistency of the rescaling estimations between the two acclimation temperatures, we can estimate the heating tolerance at the lower acclimation temperature from the heating tolerance at the higher acclimation temperature (Equation S 6). Equation 8 in the main text and Equation S 6 converts normalised heating tolerance at one acclimation temperature into rescaled heating duration (via \(g\_\beta\)), corrects for the difference in acclimation temperature along the non-linear temperature-dependence of biological rates (via \(e^{\beta\tau}\)), and converts rescaled and biological rate-corrected heating tolerance into normalised heating tolerance at the other acclimation temperature (via \(f\_\beta\)).

\[
\Delta \tau (\tau\_{a1}) \approx f\_\beta \big(e^{\beta (\tau\_{a2} - \tau\_{a1})} g\_\beta(\Delta \tau(\tau\_{a2})) \big)
\tag{6}\]

Code

```
# Add estimations of high from low and conversely low from high acclimation temperature (Equations 8 & S6)
ARR <- ARR %>% 
  mutate(delta_tau_high_hat = f_beta_fun(exp(beta_K * (tau_a_low - tau_a_high)) * g_beta_fun(delta_tau_low)), # estimate high from low (Equation 8 in the main text)
         delta_tau_low_hat = f_beta_fun(exp(beta_K * (tau_a_high - tau_a_low)) * g_beta_fun(delta_tau_high))) # estimate low from high (Equation S6)
```

## 4.4 Figure 3

Generate Figure 3 in the main text.

Code

```
pred3 <- ggplot(data = ARR,
       mapping = aes(y = delta_tau_high_hat * Tk,
                     x = delta_tau_high * Tk)) + 
  geom_point() + 
  geom_abline(intercept = 0, slope = 1, lty = 2, alpha = 0.6) + 
  geom_smooth(method = "lm", se = FALSE, col = "darkgrey") + 
  labs(y = expression("Estimated heating tolerance (\u00B0C)"),
       x = expression("Observed heating tolerance (\u00B0C)")) + 
  scale_x_continuous(expand = c(0,0), limits = c(0, 40)) +
  scale_y_continuous(expand = c(0,0), limits = c(0, 40)) 

pred3

pred4 <- ggplot(data = ARR,
                mapping =aes(y = delta_tau_low_hat * Tk,
                             x = delta_tau_low * Tk)) + 
  geom_point() + 
  geom_abline(intercept = 0, slope = 1, lty = 2, alpha = 0.6) + 
  geom_smooth(method = "lm", se = FALSE, col = "darkgrey") + 
  labs(y = expression("Estimated heating tolerance (\u00B0C)"),
       x = expression("Observed heating tolerance (\u00B0C)")) + 
  scale_x_continuous(expand = c(0,0), limits = c(0, 40)) +
  scale_y_continuous(expand = c(0,0), limits = c(0, 40))
pred4
```

Predicting heating tolerance of the higher acclimation temperature from the lower acclimation temperature. Figure 3A in main text.

Predicting heating tolerance of the lower acclimation temperature from the higher acclimation temperature. Figure 3B in main text.

Code

```
# Calculate R2
pred_high_low <- lm(delta_tau_high_hat ~ delta_tau_high, 
                   # offset = delta_tau_high_hat, 
                   data = ARR)

pred_low_high <- lm(delta_tau_low_hat ~ delta_tau_low, 
                   # offset = delta_tau_low_hat, 
                   data = ARR)
```

The \(R^2\) of predicting the thermal tolerance of the higher acclimation temperature from the lower acclimation temperature is 93.65%, and the \(R^2\) of predicting the thermal tolerance of the lower acclimation temperature from the higher acclimation temperature is 92.05%.

---

# 5 The effect of heating rate on rescaled heating duration

Code

```
# Import and prepare Morley et al 2016
varying_rates <- read.csv("data/Morley_et_al_2016.csv") %>% 
  rename(heating_rate = 8,
         ta = 9,
         tc = 10) %>% 
   mutate(Genus = ifelse(Species == "borchgrevinki", "Pagothenia", genus)) %>% # Synonym Trematomus Pagothenia Pagothenia in ARR dataset
  mutate(Name = paste(Genus, Species), 
         experiment = "variable",
         lambda = heating_rate / Tk) %>%
  # Update phyla names
  mutate(Phyla = case_when(phyla == "mollusc" ~ "Mollusca",
                           phyla == "Fish" ~ "Chordata",
                           phyla == "Ascidians" ~ "Chordata",
                           phyla == "Crustacean" ~ "Arthropoda",
                           TRUE ~ phyla)) %>%
  group_by(Name) %>%
  distinct() %>% # remove duplicated rows
  filter(n()>1) %>% # remove species with only 1 observation
  ungroup()

# Add Acclimation Response Ratio dataset to Morley et al 2016
species_data <- ARR %>%
  # Transform the ARR dataset to long format
  pivot_longer(cols = starts_with(c("ta_", "tc_")),
               names_to = c(".value", "assay"),
               names_sep = "_") %>%
  mutate(experiment = "constant") %>% 
  # Join datasets and get occurences of species in each dataset
  full_join(.,
            varying_rates, 
            by = c("Genus", "Species", "Name", "experiment", "ta", "tc", "lambda"),
            suffix = c("_constant", "_variable")) %>% 
  # Normalise temperature
  mutate(tau_a = ta / Tk, 
         tau_c = tc / Tk,
         delta_tau = tau_c - tau_a) %>% 
  group_by(Name) %>% 
  mutate(occurrence = length(unique(experiment))) %>%
  ungroup() %>% 
  # Keep species with more than 1 heating rate from either dataset
  filter(experiment == "variable" | occurrence == 2) %>% 
  # Calculate rescaled heating duration (Equation 8)
  mutate(delta_tr = pmap_dbl(list(tau_a = tau_a,
                                  tau_c = tau_c,
                                  lambda = lambda),
                             delta_tr_fun))
```

To examine the relationship between heating rate and rescaled heating duration further, we tested a series of hypotheses describing how heating rate was expected to affect the rescaling of heating tolerances. Since the Acclimation Response Ratio dataset does not contain observations made under different heating rates from the same starting temperature for a species, we used a second dataset containing upper thermal tolerances measured from the same starting temperature under different heating rates (\(\lambda\)) (Morley et al. 2016). Doing so allowed us to examine the effect of varying heating rate while keeping acclimation or starting temperature the same. We removed species with only one observation were removed (n = 3), resulting in a dataset with 107 observations from 7 phyla. *Trematomus borchgrevinki* was renamed to *Pagothenia borchgrevinki* because *Pagothenia* is the accepted genus and is also used in constant heating rates dataset. We combined this dataset (`varying_rates`) with data from the constant heating rate experiments for species that also had variable heating rate data with them (129 observations from 37 species, called `species_data`). The datasets are identified by `experiment` (`variable` or `constant`) and `occurrence` and some species occurred in both datasets.

---

## 5.1 Testing a null effect of heating rates on rescaled heating duration

First we tested a null hypothesis that rescaled heating duration does not depend on heating rate. For any heating rate:

\[
\Delta \tau (\lambda)= f\_\beta (e^{-\beta \tau\_{a}} \lambda \Delta t\_r(\lambda))
\tag{7}\]

If the null hypothesis is supported (Equation S 7), then \(\Delta t\_r (\lambda) = \Delta t\_r (0)\) and thus Equation S 7 becomes Equation 9 in the main text (below).

\[
\Delta \tau (\lambda) = f\_\beta (e^{-\beta \tau\_{a}} \lambda \Delta t\_r (0))
\]

which is a logarithmic, temperature-dependent relationship between observed heating tolerance and heating rate. Equation 9 can be tested by conducting ramping assays starting at the same acclimation temperature but using different heating rates. From these data we can infer \(\Delta t\_r (0)\) from the experiment with the slowest heating rate \(\lambda\_{min}\). We should then have that

\[
\Delta \tau (\lambda) = f\_\beta (\hat{\lambda}), \ \text{where} \ \hat{\lambda} = \frac{\lambda}{\lambda\_{min}} g\_\beta (\Delta \tau (\lambda\_{min}))
\tag{8}\]

In a plot of normalised heating tolerance (\(\Delta \tau\)) against the transformed variable \(f\_\beta (\hat{\lambda})\), the null hypothesis (Equation S 8) is supported if the points fall along the identity (1:1) line. If points systematically fall below the 1:1 line, then the null hypothesis is not supported (Figure 4A in main text).

Code

```
# Calculate ratios of Equation S8 and Figure 4A
rate_ratios <- species_data %>% 
  # Group by name
  group_by(Name) %>% 
  # For each species create all combinations of heating rate with covariates
  group_modify(~expand_grid(.x, 
                            select(.x, lambda, delta_tr, delta_tau, tau_a),
  # Add numbered suffix to all columns; unique for duplications
                            .name_repair = function (x) ave(x, x, FUN = function(i) str_c(i, seq_along(i))))) %>%
  # Calculate ratio of rescaled duration and heating rate (Equation 19)
  mutate(lambda_ratio = lambda2/lambda1,
         t_ratio = delta_tr2/delta_tr1) %>%
  # Calculate scaling coefficient gamma for each species (Equation 19)
  group_modify(function(x, y){
    x$gg <- abs(as.numeric(coef(lm(log(t_ratio) ~ log(lambda_ratio), x))[2]))
    return(x)}) %>% 
  distinct() %>% # Remove duplications created by expand grid
  ungroup() %>% 
  # Calculate estimated normalised heating tolerance at the second acclimation temperature, corrected for heating rate (Equation 20)
  mutate(delta_tau2_hat = f_beta_fun(exp(beta_K * (tau_a1-tau_a2)) * (lambda_ratio ^ (1 - gg)) * g_beta_fun(delta_tau1)),
         delta_tau2_hat_inter = f_beta_fun(exp(beta_K * (tau_a1-tau_a2)) * (lambda_ratio ^ (1 - 0.7656548)) * g_beta_fun(delta_tau1)))

# Get interspecific gamma
gamma <- abs(coef(glm(log(t_ratio) ~ log(lambda_ratio), data = rate_ratios))[2])
```

## 5.2 Figure 4A

We used the slowest heating rate of each species to test whether heating rate had no effect on rescaled heating duration and thus estimated heating tolerance. The null hypothesis was not supported because points systematically fall below the 1:1 line (Figure 4A in main text).

Code

```
g_departure <- species_data %>%
  filter(experiment == "variable") %>% 
  group_by(Name) %>% 
  mutate(lambda_min = min(lambda),
         delta_tau_min = delta_tau[which.min(lambda)]) %>% 
  ungroup() %>% 
  ggplot(mapping = aes(x = f_beta_fun(lambda / lambda_min * g_beta_fun(delta_tau_min)) * Tk,
                       y = delta_tau * Tk,
                       col = Name)) + 
  geom_point() + 
  geom_line(alpha = 0.5) +
  geom_abline(slope = 1, intercept = 0, lty = 2, alpha = 0.6) +
  labs(x = expression("Estimated heating tolerance (\u00B0C)"), 
       y = expression("Observed heating tolerance (\u00B0C)")) +
  scale_colour_viridis_d(option = "C", end = 0.9) +
  scale_x_continuous(expand = c(0,0), limits = c(0, 100)) +
  scale_y_continuous(expand = c(0,0), limits = c(0, 25)) +
  theme(legend.position="none", plot.margin = margin(5.5, 7, 5.5, 5.5, unit = "pt"))

g_departure
```

Estimateed heating tolerance versus observed heating tolerance for species with varying heating rates (107 observations). Colours are species (n = 37). Figure 4A in main text.

## 5.3 Figure 4B

Code

```
g_scaling <- ggplot(data = rate_ratios,
                    mapping = aes(x = lambda_ratio, 
                                  y = t_ratio, 
                                  group = Name,
                                  col = Name)) + 
  geom_line(stat ="smooth", method = "lm", alpha = 0.5) +
  geom_point() + 
  geom_abline(slope = -1, intercept = 0, lty = 2, alpha = 0.6) +
  geom_smooth(aes(group = 1), method = "lm", se = FALSE, col = 1) +
 scale_x_log10(
   breaks = scales::trans_breaks("log10", function(x) 10^x),
   labels = scales::trans_format("log10", scales::math_format(10^.x))
 ) +
 scale_y_log10(
   breaks = scales::trans_breaks("log10", function(x) 10^x),
   labels = scales::trans_format("log10", scales::math_format(10^.x))
 ) +
  ggtitle(paste0("slope = ", 
                 round(gamma, 2))) +
  labs(x = "Heating rate ratio", y = "Rescaled heating duration ratio") +
  scale_colour_viridis_d(option = "C", end = 0.9) +
  theme(legend.position="none")

g_scaling
```

Interspecific scaling of rescaled heating duration with heating rate. Variation within each species is shown by the fitted lines between points (129 observations). The dashed identity line is the expected relationship following the null hypothesis. Axes are Log10 transformed. Colours are species (n = 37). Figure 4B in main text.

To characterise the effect of heating rate on rescaled heating duration, we plot the ratio of rescaled heating duration for a paired observation within a species with the corresponding ratio for paired heating rate. The relationship between the ratio of rescaled heating duration and the ratio of heating rate is described by an allometric scaling equation in the form \(\Delta t\_r (\lambda) \sim c \lambda^{- \gamma}\) where \(c\) depends only on the experimental protocol and the species-specific scaling exponent, \(\gamma\). Points should fall along the identity line if heating rate does not affect rescaled heating duration (i.e., a scaling exponent of -1).

Change in rescaled heating duration, and thus heating tolerance, scales with change in heating rate according to an allometric scaling equation. There is some species-specific variation in the slope of the regression \(\gamma\) (colours). We can calculate an interspecific scaling exponent of \(\gamma =\) 0.76 calculated from a linear regression of ratios of heating rate and ratios of rescaled heating duration on Log10 scales (Figure 4B in main text).

---

## 5.4 Quantifying the effect of heating rates on rescaled heating duration

Since higher heating rates reduces rescaled heating duration following a phenomenological power law, then the relationship between two experiments done at two heating rates, \(\lambda\_1\) and \(\lambda\_2\), regardless of the starting temperature, can be described by:

\[
\frac{\lambda\_2 \Delta t\_r (\lambda\_2)}{\lambda\_1 \Delta t\_r (\lambda\_1)} \approx \bigg(\frac{\lambda\_2}{\lambda\_1}\bigg)^{1-\gamma}
\tag{9}\]

and therefore that from one heating tolerance \(\Delta \tau\_1\) we should be able to estimate any other \(\Delta \tau\_2\). Using Equation S 9 we can deduce \(\Delta t\_r (\lambda\_2)\) and from Equations 6 and 7 leads to Equation 10 in the main text:

\[
\Delta\tau\_2 = f\_{\beta}\Bigg(e^{\beta(\tau\_{a1}-\tau\_{a2})}\bigg(\frac{\lambda\_2}{\lambda\_1}\bigg)^{1-\gamma}g\_{\beta}\big(\Delta\tau\_1\big)\Bigg)
\]

That is, we can estimate one unknown heating tolerance \(\Delta\tau\_2\) from another known value \(\Delta\tau\_1\) using Equation 10 in the main text (above) which incorporates the correction for the effect of heating rate on rescaled heating duration using a species-specific scaling exponent (\(\gamma\)). We use species-specific scaling exponents in subsequent calculations for precision rather than the interspecific scaling exponent. Doing so, we see strong concordance of the observed heating tolerance (`delta_tau2`) and the estimated value (`delta_tau2_hat`).

## 5.5 Figure 4C

Code

```
g_estimate_tau <- rate_ratios %>% 
  filter(t_ratio != 1 & lambda_ratio != 1) %>% # remove trivial pairs where it is the lowest heating rate
  ggplot(mapping = aes(x = delta_tau2_hat * Tk, 
                       y = delta_tau2 * Tk,
                       col = Name)) + 
  geom_abline(slope = 1, intercept = 0, lty = 2, alpha = 0.6) +
  geom_point() + 
  geom_smooth(method = "lm", se = FALSE, col = 1) + 
  labs(x = expression("Estimated heating tolerance (\u00B0C)"), 
       y = expression("Observed heating tolerance (\u00B0C)")) +
  scale_colour_viridis_d(option = "C", end = 0.9) +
  scale_x_continuous(expand = c(0,0), limits = c(0, 30)) +
  scale_y_continuous(expand = c(0,0), limits = c(0, 30)) +
  theme(legend.position="none")

g_estimate_tau
```

Estimated heating tolerance corrected using species-specific scaling exponents for the allometric relationship between rescaled heating duration and heating rate. Colours are species. Figure 4C in main text.

Code

```
# R2 of the scaling relationship using species-specific values of gamma
species_gamma <- rate_ratios %>% 
   filter(t_ratio != 1 & lambda_ratio != 1) %>% 
  lm(delta_tau2_hat ~  delta_tau2, .) %>% 
  summary(.) %>%
  .$r.squared %>% 
  round(., 4) * 100
```

The \(R^2\) is 87.9%.

## 5.6 Interspecific scaling exponent of \(\gamma\)

Code

```
# R2 of the scaling relationship using the interspecific value of gamma
interspecific_gamma <- rate_ratios %>% 
   filter(t_ratio != 1 & lambda_ratio != 1) %>% 
  lm(delta_tau2_hat_inter ~  delta_tau2, .) %>% 
  summary(.) %>%
  .$r.squared %>% 
  round(., 4) * 100
```

We plotted the same as Figure 4C but using the interspecific value of 0.76 for \(\gamma\). Doing so increases our estimation error for a species (Figure S 4). The \(R^2\) is 54.51%.

Code

```
rate_ratios %>% 
   filter(t_ratio != 1 & lambda_ratio != 1) %>% # remove trivial pairs where it is the lowest heating rate
  ggplot(mapping = aes(x = delta_tau2_hat_inter * Tk, 
                       y = delta_tau2 * Tk,
                       col = Name)) + 
  geom_abline(slope = 1, intercept = 0, lty = 2, alpha = 0.6) +
  geom_point() + 
  geom_smooth(method = "lm", se = FALSE, col = 1) + 
  scale_x_continuous(expand = c(0,0), limits = c(0, 30)) +
  scale_y_continuous(expand = c(0,0), limits = c(0, 30)) +
  labs(x = expression("Estimated heating tolerance (\u00B0C)"), 
       y = expression("Observed heating tolerance (\u00B0C)")) +
  scale_colour_viridis_d(option = "C", end = 0.9) +
  theme(legend.position="none")
```

Figure 4: Estimated heating tolerances calculated using a value of 0.76 for gamma, representing an interspecific parameter. Colours are species.

---

# 6 Generalised graphs of rescaled heating durations

We visualised the generalised relationship described by Equation 10 in the main text, using median values of heating rate, upper thermal tolerance and acclimation temperature across all species and the interspecific scaling exponent, \(\gamma =\) 0.76.

Code

```
sp_heat_rate = median(ARR$heating_rate)
sp_ta = median(ARR$ta_low)
sp_tc = median(ARR$tc_low)

heating_rate <- round(sort(c(10^(seq(log10(0.004), log10(60), length.out = 14)), 0.015, 1, 0.042, 18)), 3) # on log10 scale for equidistant points
heating_rate <- heating_rate[!heating_rate %in% c(0.018, 0.037, 13.667)] # neaten lines
ta <- seq(5, 40, 1) # range of acclimation temperatures
pal_cols <- viridisLite::viridis(n = length(heating_rate), end = 0.9) # colour palette for points

gen_ecto <- expand_grid(heating_rate, ta) %>% 
  mutate(sp_lambda = sp_heat_rate / Tk,
         sp_tau_a = sp_ta / Tk,
         sp_tau_c = sp_tc / Tk,
         sp_delta_tau = sp_tau_c - sp_tau_a,
         tau_a = ta / Tk, 
         delta_tau_a =  sp_tau_a - tau_a,
         lambda = heating_rate / Tk,
         lambda_ratio = lambda/sp_lambda)  %>% 
  mutate(delta_tau_hat =  f_beta_fun(exp(beta_K * delta_tau_a) * (lambda_ratio ^ (1 - gamma)) * g_beta_fun(sp_delta_tau))) %>% 
  ggplot() + 
  geom_line(aes(ta, delta_tau_hat * Tk, group = heating_rate, col = heating_rate)) +
  labs(x = expression("Acclimation temperature (\u00B0C)"), 
       y = expression("Expected heating tolerance (\u00B0C)")) +
  scale_colour_viridis_c(name = expression("Heating rate (\u00B0C h" ^-1 * ")"), end = 0.9, trans = "log10", labels = function(x) signif(x)) 

gen_ecto
```

Figure 5: Generalised graph of estimated heating tolerance by acclimation temperature and heating rate (colours).

## 6.1 Figure 5

Figure S 5 can also be visualised as estimated upper thermal tolerance limit \(T\_c\) for any acclimation temperature \(T\_a\) .

Code

```
fig5 <- expand_grid(heating_rate, ta) %>% 
  mutate(sp_lambda = sp_heat_rate / Tk,
         sp_tau_a = sp_ta / Tk,
         sp_tau_c = sp_tc / Tk,
         sp_delta_tau = sp_tau_c - sp_tau_a,
         tau_a = ta / Tk, 
         delta_tau_a =  sp_tau_a - tau_a,
         lambda = heating_rate / Tk,
         lambda_ratio = lambda/sp_lambda)  %>% 
  mutate(delta_tau_hat =  f_beta_fun(exp(beta_K * delta_tau_a) * (lambda_ratio ^ (1 - gamma)) * g_beta_fun(sp_delta_tau))) %>% 
  ggplot() + 
  geom_line(aes(ta, delta_tau_hat * Tk + ta, group = heating_rate, col = heating_rate)) +
  labs(x = expression("Acclimation temperature (\u00B0C)"), 
       y = expression("Upper thermal tolerance limit (\u00B0C)")) +
  scale_colour_viridis_c(name = expression("Heating rate (\u00B0C h" ^-1 * ")"),
                         trans = "log10",
                         end = 0.9,
                         labels = function(x) signif(x)) 

fig5
```

Generalised graph of estimated upper thermal tolerance limit by acclimation temperature and heating rate (colours). Figure 5 in the main text.

---

# 7 Table of equations

Table 3: All numbered equations in supplementary (denoted Equation S) and main text in the order presented.

| Equation | Description |
| --- | --- |
| 1 | Heating tolerance; range of temperatures between acclimation temperature and upper thermal tolerance |
| 2 | Elapsed duration of a heating assay between the start (\(t\_a\)) and end (\(t\_c\)) time points |
| Equation S 1 | Describing the relationship between two heating durations |
| Equation S 2 | Describing the integration of the exponential temperature-dependence of biological rates between the start and end of the ramping assay |
| Equation S 3 | Deriving the \(\beta\) coefficient |
| 3 | Approximating the temperature-dependence of biological rates with the \(\beta\) coefficient, Equation S 3 |
| 4 | Simplified form of Equation S 2 using Equation S 3 describing the same relationship |
| 5 | Simplified form of Equation 4 describing the same relationship and including a constant heating rate (\(\lambda\)) |
| 6 | The integration of Equation 5 to calculate rescaled heating duration from heating tolerance on a normalised temperature scale |
| 7 | The inverse of Equation 6 to calculate heating tolerance on a normalised temperature scale from rescaled heating duration |
| Equation S 4 | The simplest hypothesis for how one known heating tolerance can be used to estimate an unknown heating tolerance at another acclimation temperature, assuming rescaled heating durations are the same |
| Equation S 5 | An alternative hypothesis for how one known heating tolerance can be used to estimate an unknown heating tolerance at another acclimation temperature, accounting for differences in acclimation temperature |
| 8 | A fully derived hypothesis for how one known heating tolerance can be used to estimate an unknown heating tolerance at another acclimation temperature, accounting for differences in acclimation temperature and the integration of the exponential temperature-dependence of biological rates. Used to estimate thermal tolerance at the higher acclimation temperature from the lower acclimation temperature |
| Equation S 6 | The same as Equation 8 to estimate thermal tolerance at the lower acclimation temperature from the higher acclimation temperature |
| Equation S 7 | General expression describing that heating rate has no effect on rescaled heating duration |
| 9 | Extension of Equation S 7 describing the null hypothesis that heating rate has no effect on rescaled heating duration |
| Equation S 8 | Extension of Equation 9 describing the null hypothesis that heating rate has no effect on rescaled heating duration based on comparing the ratio of the slowest heating rate with all other rates measured for a species |
| Equation S 9 | General description of the power scaling relationship between two assays measured using two heating rates |

---

# 8 Manuscript Figures

Code

```
# rescaled graphs
legend <- get_legend(heating_graph + theme(legend.box.margin = margin(0, 0, 0, 0)))

p2 <- plot_grid(heating_graph + theme(legend.position="none"),
                rescaled_duration + theme(legend.position="none"),
                # rescaled_tolerances + theme(legend.position="none"),
                labels = "AUTO", nrow = 1, align = "hv", hjust = -1, label_size = 10)

fig2 <- plot_grid(p2, legend, rel_widths =c(3, 0.5))
fig2

# estimation graphs
pred_high_low <- pred3 + aes(col = heating_rate) + 
  scale_x_continuous(expand = c(0,0), limits = c(0, 40)) +
  scale_y_continuous(expand = c(0,0), limits = c(0, 40)) +
  geom_smooth(method = "lm", se = FALSE, col = 1) +
  scale_colour_viridis_c(name = expression("Heating rate (\u00B0C h" ^-1 * ")"), end = 0.9, trans = "log10", labels = function(x) signif(x)) +
  theme(legend.position="none")

pred_low_high <- pred4 + aes(col = heating_rate) + 
  scale_x_continuous(expand = c(0,0), limits = c(0, 40)) +
  scale_y_continuous(expand = c(0,0), limits = c(0, 40)) +
  geom_smooth(method = "lm", se = FALSE, col = 1) +
  scale_colour_viridis_c(name = expression("Heating rate (\u00B0C h" ^-1 * ")"), end = 0.9, trans = "log10", labels = function(x) signif(x)) +
  theme(legend.position="none")

p3 <- plot_grid(pred_high_low, pred_low_high, labels = "AUTO", nrow = 1, align = "h", hjust = -1, label_size = 10)
fig3 <- plot_grid(p3, legend, rel_widths =c(3, 0.5))
fig3

# Heating rate effect
fig4 <- plot_grid(g_departure, g_scaling, g_estimate_tau, labels = "AUTO", nrow = 1, align = "h", hjust = -1, label_size = 10)
fig4
```

Figures saved as TIFF. Resulting dataset containing all analyses (`ARR`) are saved as CSV.

Code

```
journal <- "JAnim"
dir.create(paste0("../figures/", journal, "/"))

for(i in c("tiff", "jpeg")){
save_plot(plot = fig2, filename = paste0("../figures/", journal, "/fig2.", i), dpi = 300, base_width = 10, bg = "white")
save_plot(plot = fig3, filename = paste0("../figures/", journal, "/fig3.", i), dpi = 300, base_width = 10, bg = "white")
save_plot(plot = fig4, filename = paste0("../figures/", journal, "/fig4.", i), dpi = 300, base_width = 8, base_height = 3, bg = "white")
save_plot(plot = fig5, filename = paste0("../figures/", journal, "/fig5.", i), dpi = 300, base_width = 5, bg = "white")
}

# Supplementary
save_plot(plot = sensi_plot, filename = paste0("../figures/", journal, "/figS1.jpeg"), dpi = 300, base_width = 10, base_height = 5, bg = "white")
# save_plot(plot = rescaled_tolerances, filename = paste0("../figures/", journal, "/figS2.jpeg"), dpi = 300, bg = "white")
save_plot(plot = pred1, filename = paste0("../figures/", journal, "/figS3.jpeg"), dpi = 300, bg = "white")
save_plot(plot = pred2, filename = paste0("../figures/", journal, "/figS4.jpeg"), dpi = 300, bg = "white")

# data
write.csv(ARR, file = "output/rescaled_heating_tolerances_data.csv", row.names = FALSE)
```

---

# 9 Session information

```
R version 4.4.2 (2024-10-31)
Platform: aarch64-apple-darwin20
Running under: macOS Sequoia 15.3.1

Matrix products: default
BLAS:   /Library/Frameworks/R.framework/Versions/4.4-arm64/Resources/lib/libRblas.0.dylib 
LAPACK: /Library/Frameworks/R.framework/Versions/4.4-arm64/Resources/lib/libRlapack.dylib;  LAPACK version 3.12.0

locale:
[1] en_US.UTF-8/en_US.UTF-8/en_US.UTF-8/C/en_US.UTF-8/en_US.UTF-8

time zone: Europe/Dublin
tzcode source: internal

attached base packages:
[1] stats     graphics  grDevices utils     datasets  methods   base     

other attached packages:
 [1] cowplot_1.1.3   lubridate_1.9.4 forcats_1.0.0   stringr_1.5.1  
 [5] dplyr_1.1.4     purrr_1.0.2     readr_2.1.5     tidyr_1.3.1    
 [9] tibble_3.2.1    ggplot2_3.5.1   tidyverse_2.0.0

loaded via a namespace (and not attached):
 [1] generics_0.1.3    stringi_1.8.4     lattice_0.22-6    hms_1.1.3        
 [5] digest_0.6.37     magrittr_2.0.3    evaluate_1.0.3    grid_4.4.2       
 [9] timechange_0.3.0  fastmap_1.2.0     cellranger_1.1.0  jsonlite_1.8.9   
[13] Matrix_1.7-1      mgcv_1.9-1        viridisLite_0.4.2 scales_1.3.0     
[17] cli_3.6.3         rlang_1.1.4       munsell_0.5.1     splines_4.4.2    
[21] withr_3.0.2       yaml_2.3.10       tools_4.4.2       tzdb_0.4.0       
[25] colorspace_2.1-1  vctrs_0.6.5       R6_2.5.1          lifecycle_1.0.4  
[29] htmlwidgets_1.6.4 pkgconfig_2.0.3   pillar_1.10.1     gtable_0.3.6     
[33] glue_1.8.0        xfun_0.50         tidyselect_1.2.1  rstudioapi_0.17.1
[37] knitr_1.49        farver_2.1.2      htmltools_0.5.8.1 nlme_3.1-166     
[41] rmarkdown_2.29    labeling_0.4.3    compiler_4.4.2    readxl_1.4.3
```

---

## 10 References

Dell, Anthony I., Samraat Pawar, and Van M. Savage. 2011. “Systematic Variation in the Temperature Dependence of Physiological and Ecological Traits.” Journal Article. *Proceedings of the National Academy of Sciences* 108 (26): 10591–96. https://doi.org/10.1073/pnas.1015178108.

Morley, S. A., A. E. Bates, M. Lamare, J. Richard, K. D. Nguyen, J. Brown, and L. S. Peck. 2016. “Rates of Warming and the Global Sensitivity of Shallow Water Marine Invertebrates to Elevated Temperature.” Journal Article. *Journal of the Marine Biological Association of the United Kingdom* 96 (1): 159–65. https://doi.org/10.1017/S0025315414000307.

Morley, S. A., L. S. Peck, J. M. Sunday, S. Heiser, and A. E. Bates. 2019. “Physiological Acclimation and Persistence of Ectothermic Species Under Extreme Heat Events.” Journal Article. *Global Ecology and Biogeography* 28 (7): 1018–37. https://doi.org/10.1111/geb.12911.

Morley, S. A., L. S. Peck, J. Sunday, S. Heiser, and A. E. Bates. 2018. “Acclimation Potential of Global Ectothermic Species, Collated from Literature, 1960 to 2015.” Dataset. Natural Environment Research Council, Cambridge, UK. https://doi.org/https://doi.org/10.5285/20010bfb-c6d3-430f-b1f7-d16790ab8359.

R Core Team. 2022. *R: A Language and Environment for Statistical Computing*. Vienna, Austria: R Foundation for Statistical Computing. https://www.R-project.org/.

Wickham, Hadley. 2019. *Tidyverse: Easily Install and Load the Tidyverse*. https://CRAN.R-project.org/package=tidyverse.

Wilke, Claus O. 2020. *Cowplot: Streamlined Plot Theme and Plot Annotations for ’Ggplot2’*. https://CRAN.R-project.org/package=cowplot.


##### Source Code

```
---
title: "Heat limits scale with metabolism in ectothermic animals"
subtitle: "Supplementary material"
project:
    execute-dir: project
date: last-modified
author:
  - name: Jacinta Kong
    email: jacintadkong@gmail.com
    affiliations:
    - name: Trinity College Dublin, Ireland
  - name: Andrew L Jackson
    email: jacksoan@tcd.ie
    affiliations:
    - name: Trinity College Dublin, Ireland
  - name: Nicholas Payne
    email: paynen@tcd.ie
    affiliations:
    - name: Trinity College Dublin, Ireland
  - name: Jean Fran&ccedil;ois Arnoldi
    email: arnoldi.jeff@gmail.com
    affiliations:
    - name: Trinity College Dublin, Ireland    
  
toc: true
number-sections: true
bibliography: rates.bib
editor_options:
  chunk_output_type: console
format: 
    html:
        page-layout: full
        theme: litera
        embed-resources: true
        code-fold: show
        code-tools: true
        code-block-border-left: true
        code-block-background: false
        highlight-style: github
crossref:
  fig-prefix: Figure S
  tbl-prefix: Table S
  eq-prefix: Equation S
---

```{r setup, message=FALSE, echo = FALSE}
knitr::opts_chunk$set(
  message = FALSE,
  warning = FALSE,
  comment = NA,
  echo = TRUE,
  fig.align = 'center',
  fig.width = 6,
  fig.height = 5
)

library(tidyverse)
library(cowplot)
theme_set(theme_classic(base_size = 10)) # set default font size
options(tibble.width = Inf)
```

**README**  
This document presents the full workflow and code of *Heating tolerance of ectotherms scales with temperature’s non-linear influence on biological rates*, including data processing and generating figures for the study. The analysis was conducted in `r R.version.string` [@R-base] running `Tidyverse` [@R-tidyverse] & `cowplot` [@R-cowplot] (@sec-session). Supplementary analysis and notation are presented here. See Methods in main text for the primary mathematical notation and derivation, and @tbl-equations for the order of equations. 

***

# Heating tolerance data

```{r}
# Import Morley et al 2018
ARR <- readxl::read_excel("data/Morley_et_al_(2018)_-_Collated_Species_Acclimation_Data.xlsx",  skip = 8) %>% 
  # Add unique id
  rowid_to_column("row_ID") %>%  
  # remove blank rows
  filter(!is.na(Taxon)) %>%
  # rename columns
  rename("ta_high" = 11, #"High acclimation Temperature /°C" ,
         "ta_low" = 12, #"Low Acclimation Temperature/ °C" ,
         "ARR" = 17, #"CTMax ARR" ,
         "tc_high" = 16, #"CTMax/ °C", # CT max of the high acclimation temperature
         "acclim_window" = 13, #"Acclimation Window /°C",
         "heating_rate" = 22, #"°C change hr-1",
         "collection_latitude" = 19) %>%  #"Latitude of Collection/ °") %>%
  # Fix typo in taxon
  mutate(Taxon = str_replace_all(Taxon, "amphibains", "amphibians")) %>%
  # Fix incorrect heating rate in DOI 10.1007/s10695-013-9793-7
  mutate(heating_rate = ifelse(row_ID  == 367, (0.3 * 60), heating_rate)) %>%
  # Calculate low temperature CT max
  mutate(tc_low = tc_high - (ARR * acclim_window)) 


# Calculate heating tolerances for each acclimation temperature, H (Equation 1)
ARR <- ARR %>% 
  mutate(ht_low = tc_low - ta_low,
         ht_high = tc_high - ta_high)
```

We reanalyzed a dataset on thermal tolerances of diverse ectotherms containing Acclimation Response Ratios (ARR), acclimation temperatures ($T_a$) and upper thermal tolerance limits ($T_c$) compiled from the literature between 1960 – 2015 [@Morley2018;@Morley2019]. The original dataset is available from the [Polar Data Centre](https://doi.org/10.5285/20010bfb-c6d3-430f-b1f7-d16790ab8359) under an Open Government Licence v3.0 [@Morley2018]. Specifically the file is named "Morley et al (2018) - Collated Species Acclimation Data.xlsx" on the repository but the downloaded file has white space replaced with "_". Ectotherms were acclimated for between `r min(ARR$'Acclimation duration/ days')` and `r max(ARR$'Acclimation duration/ days')` days to a lower temperature or a higher temperature for a paired observation for each species. 

Data were only included if each species had thermal tolerances measured for two different starting acclimation temperatures with an average of 10 &deg;C apart, allowing us to compare heating tolerances both within and among species. The dataset contained `r nrow(ARR)` observations of `r length(unique(ARR$Name))` species from `r length(unique(ARR$Class))` Classes from 7 Phyla: Chordata, Arthropoda, Mollusca, Platyhelminthes, Echinodermata, Brachiopoda and Cnidaria). These species spanned northern and southern hemispheres, polar to tropical latitudes, and freshwater, marine and terrestrial habitats.

Animals were heated under a constant ramping temperature from their acclimation temperature until the point of organismal collapse, which we refer to here as their upper thermal tolerance limit ($T_c$), called CT~max~ in @Morley2019. A small proportion of observations used lethal temperatures as the endpoint and we did not exclude these observations but do not consider our analysis to appropriately apply to mortality rates or liklihood of survival. Within paired observations, each species was measured at the same constant heating rate, and heating rates varied among paired observations representing independent species or experimental assays. Thermal tolerance limits were assayed for both acclimation temperatures in paired observations for a species (referred as `tc_low` or `tc_high` in this code). 

The upper thermal tolerance limit of the lower acclimation temperature was not reported in @Morley2019. We derived the upper thermal tolerance limit of the lower acclimation temperature from rearranging the formula for Acclimation Response Ratios. We first multiplied the acclimation response ratio for each species by the difference in acclimation temperatures; called acclimation window (`acclim_window`) following @Morley2019. We then subtracted this result from the upper thermal tolerance of the higher acclimation temperature (`tc_high`) for each species to calculate the upper thermal tolerance of the lower acclimation temperature (`tc_low`).

$$
T_{c \space lower} = T_{c \space higher} - (ARR \times acclimation \space window)
$$

## Figure 2A

Generate Figure 2A of heating tolerance.

```{r}
#| fig-cap: "Heating tolerances at the lower and higher acclimation temperatures for paired observations on Log 10 transformed axes. Figure 2A in main text."

heating_graph <- ARR %>% 
  ggplot(aes(ht_low, ht_high, col = heating_rate)) +
  geom_abline(slope = 1, intercept = 0, lty = 2, alpha = 0.6) +
  geom_smooth(method = "lm", se = FALSE, col = 1) + 
  geom_point() +
  labs(x = expression("Lower acclimation temperature H (\u00B0C)"),
       y = expression("Higher acclimation temperature H (\u00B0C)"),
       title = expression("Heating tolerance, H")) +
  scale_x_continuous(limits = c(3,40), expand = c(0,0), trans = "log10") +
  scale_y_continuous(limits = c(3,40), expand = c(0,0), trans = "log10") +
  coord_equal() +  
  scale_colour_viridis_c(name = expression("Heating rate (\u00B0C h" ^-1 * ")"), end = 0.9, trans = "log10", labels = function(x) signif(x))

heating_graph
```
***

# Rescaling heating tolerance via heating duration

## Data requirements

Rescaling heating tolerances requires an assay of thermal limits using a ramping protocol with a constant heating rate. The required variables are upper thermal tolerance limit ($T_c$), acclimation temperature or the starting temperature of the ramping assay ($T_a$), and heating rate of the ramping assay (heating rate). These variables are used to calculate heating tolerance and elapsed duration (heating duration) of the ramping assay. Acclimation and starting temperature are used interchangeably as acclimation temperature is the starting temperature of the heating assay in our dataset. @tbl-notation contains a summary of the main mathematical notation used.

Our calculations can be generalised to any heating event with a constant heating rate or a known duration of time at a constant temperature. Our model does not formally apply to other protocols of assaying thermal tolerance limits or to lower thermal tolerance limits but these variables present avenues for future studies. Rescaling heating duration and heating tolerance is dependent on three assumptions: 1) Heating tolerance is arbitrarily defined with respect to 0 &deg;C, 2) heating rate is constant, and 3) the Universal Temperature Dependence (Boltzmann-Arrhenius equation) is not affected by the physiological state of the organism or the experimental conditions (i.e., activation energy ($E$) does not change).

## Describing non-linear relationship between biological rates and temperature

Heating tolerances represent a duration of time (heating duration, $\Delta t$) between two time points ($t_a$, the start of the heating assay, and $t_c$, the end of the heating assay) that have a corresponding temperature (acclimation temperature, $T_a$ and upper thermal tolernace $T_c$, respectively). 

Therefore, as we can expect biological processes occurring during heating to scale with temperature we can also expect the durations of theses biological processes at one temperature, $\Delta t(T)$, to scale with temperature. We can describe the relationship between heating durations starting from two temperatures ($T_{a1}$ and $T_{a2}$) as:

$$
\frac{\Delta t(T_{a1})}{\Delta t(T_{a2})} \approx e^{\frac{E}{kT_{a1}}-\frac{E}{kT_{a2}}}
$$ {#eq-S1}

In @eq-S1, $\Delta t$ are elapsed durations of the heating assay starting at the acclimation temperature, $E$ is the activation energy of the Universal Temperature Dependence (0.66 eV) and $k$ is Boltzmann’s constant. Following @eq-S1, we can use a reference temperature to normalize heating durations to a normalized temperature scale ($\tau$). We use 0 &deg;C as the reference temperature and define a normalization constant ($T_K$) with a value of 273.15 (i.e. 0°C in Kelvin) for centering temperatures ($T$) on the Celsius scale.

$$
\tau = \frac{T}{T_K} 
$$
Here, $T$ are temperatures on the Celsius scale and $\tau$ are normalised temperatures that are centred around the normalisation constant $T_K$. Normalised temperature $T_K$ is unitless and can be converted back into degree Celsius by $T=\tau \times T_K$.

```{r}
# Constants in Equation S1
# Boltzmann's constant, k, in units of eV K-1
k <- 8.61733 * 10 ^ -5
# Normalisation constant to center around 273.15K (T_K)
Tk <- 273.15
```


Substituting the normalisation constant $T_K$ as $T_{a1}$ and temperature $T$ (in &deg;C) as $T_{a2}$ in @eq-S1, we can derive an initial expression that rescales heating durations by accounting for acclimation temperature: 

$$\Delta t_r = e^{\frac{E}{kT_K}}e^{-\frac{E}{kT}} \Delta t(T)$$
Subscript $r$ denotes variables rescaled by the non-linear temperature dependence of biological rates (@tbl-notation). $\Delta t_r$ are rescaled heating durations that correspond to non-rescaled heating duration ($\Delta t$).

Rescaled heating duration can be written as an integral between the start ($t_a$) and end times ($t_c$) of the heating assay, assuming a constant temperature ramp:

$$
\Delta t_r = e^{\frac{E}{kT_K}} \int_{t_a}^{t_c} e^{-\frac{E}{kT(t)}} dt
$$ {#eq-S2}

We can simplify @eq-S2 by using the normalised temperature scale ($\tau$) which removes the dimension of temperature. On a normalised temperature scale ($\tau$), $\frac{E}{kT}$ in @eq-S2 becomes:

$$
\frac{E}{kT} = {\frac{E}{kT_K}} {\frac{1}{1 + \frac{T}{T_K}}} \\
 = {\frac{E}{kT_K}} {\frac{1}{1 + \tau}} \\
 \approx \beta (1-\tau)
$$ 

Where 

$$
\beta = \frac{E}{k T_K}
$$ {#eq-betaS3}

```{r}
# define \beta for a given activation energy, E = 0.66 (Equation S3)
beta_K <- 0.66 / (k * Tk)
```

$\beta$ is a constant constructed from activation energy $E$, Boltzmann’s constant $k$, and the reference temperature $T_K$ with an approximate value of 28 using $E =$ 0.66 (`beta_K`) (@eq-betaS3). Following Equation 3 in the main text, we can use @eq-betaS3 to approximate the non-linear thermal dependence of biological rates and derive Equations 4 and 5 in the main text for calculating rescaled heating durations accounting for heating rate and non-linear rate-time relationships.

## Defining functions and notation
Define functions from main text to rescale heating duration. Equation 6 uses both `delta_tr_fun` and `g_beta_fun`.
```{r}
# function g of x and the constant beta (Equation 6)
g_beta_fun <- function(x){(exp(beta_K * x) - 1) / beta_K}

# function for delta t_r that uses g_beta_fun (Equation 6)
delta_tr_fun <- function(tau_a, tau_c, lambda){
  exp(beta_K * tau_a) / lambda * g_beta_fun(tau_c - tau_a)
}

# function f of x and the constant beta (Equation 7)
f_beta_fun <- function(x){log(1 + beta_K * x) / beta_K}
```

| Symbol | Description |
|:------:|:------------|
| $H$ | Heating tolerance; range of temperatures between acclimation temperature ($T_a$) and upper thermal tolerance ($T_c$)    | 
| $\Delta t$ | Elapsed duration of a heating assay between the start ($t_a$) and end ($t_c$) time points    |
| $_r$  | Variables rescaled by the temperature-dependence of biological rates  |
| $_a$  | Variables referring to acclimation or starting temperature of a heating assay |
| $_c$  | Variables referring to the upper thermal tolerance limit of a heating assay   |
| $\tau$ | Normalised temperature scale centred around $T_K$ (normalisation constant `r Tk`K) | 
| $\lambda$ | Normalised heating rate centred around $T_K$ | 
| $\gamma$ | Scaling exponent for the effect of heating rate $\lambda$ on rescaled heating duration $\Delta t_r$    | 

: Main mathematical notation and description used in this analysis. {#tbl-notation}

## Code to rescale heating tolerances

```{r}
# Step 1. Normalise acclimation temperature, upper thermal tolerances and heating rate by the normalisation constant (Tk, called tau_*), then calculate normalised heating tolerance (called delta_tau_*)
ARR <- ARR %>% 
  mutate(
    # acclimation temperatures
    tau_a_low = ta_low / Tk,
    tau_a_high = ta_high / Tk,
    # upper thermal tolerances
    tau_c_low = tc_low / Tk,
    tau_c_high = tc_high / Tk,
    # heating rate
    lambda = heating_rate / Tk,
    # normalised heating tolerance
    delta_tau_low = tau_c_low - tau_a_low,
    delta_tau_high = tau_c_high - tau_a_high)

# Step 2. Calculate rescaled heating duration for lower acclimation temperatures using Equation 6
ARR <- ARR %>% 
  mutate(delta_tr_low = pmap_dbl(list(tau_a = tau_a_low,
                                  tau_c = tau_c_low,
                                  lambda = lambda),
                             delta_tr_fun))

# Step 3. Calculate rescaled heating duration for higher acclimation temperatures using Equation 6
ARR <- ARR %>% 
  mutate(delta_tr_high = pmap_dbl(list(tau_a = tau_a_high,
                                   tau_c = tau_c_high,
                                   lambda = lambda),
                              delta_tr_fun))
```

## Figure 2B

Generate Figure 2B in the main text of rescaled heating duration.

```{r}
#| fig.cap = "Rescaled heating duration at the lower and higher acclimation temperature on Log 10 axes have a strong dependency with heating rate (colours). Figure 2B in main text."
rescaled_duration <- ggplot(data = ARR,
                            mapping = aes(x = delta_tr_low,
                                          y = delta_tr_high,
                                          col = heating_rate)) +
  geom_abline(slope = 1, intercept = 0, lty = 2, alpha = 0.6) +
  geom_smooth(method = "lm", se = FALSE, col = 1) +
  geom_point() +
  scale_x_continuous(trans = "log10", labels = function(x) round(x), expand = c(0,0), limits = c(0.1, 10000)) +
  scale_y_continuous(trans = "log10", labels = function(x) round(x), expand = c(0,0), limits = c(0.1, 10000)) +
  coord_equal() +
  labs(x = expression("Lower acclimation temperature " * Delta * t[r]),
       y = expression("Higher acclimation temperature " * Delta * t[r])) +
  ggtitle(expression(paste("Rescaled heating duration, ") * Delta * t[r])) +
  scale_colour_viridis_c(name = expression("Heating rate (\u00B0C h" ^-1 * ")"), end = 0.9, trans = "log10", labels = function(x) signif(x))

rescaled_duration
```

***

# Sensitivity of activation energy

Interspecific activation energy of various thermally-dependent traits ranges between 0.2 and 1.2 with a median of 0.55 eV and mean of 0.66eV [@Dell2011]. We investigated the sensitivity of our results to variation in activation energy across the range of activation energies reported in @Dell2011. `delta_tr_gen` is a generalised function to rescale heating tolerance with $E$ as a variable. $\beta$ values are calculated for each value of $E$. We found changing activation energy and thus $\beta$ does not meaningfully change the reported patterns (@fig-sensitivity) and $R^2$ values are above 0.9 across the range of activation energies used (@tbl-sensitivity).

```{r}
# Generalised function for rescaling heating tolerance that accepts any activation energy
delta_tr_gen <- function(x, ev, k = 8.61733*10^-5, Tk = 273.15) { # ev is activation energy
  
  # calculate beta K constant (Equation S3)
  beta_K_sensi <- ev / (k * Tk)
  
  # function for delta tr that uses g_beta_fun (Equation 6)
  delta_tr_gen <- function(tau_a, tau_c, lambda){
    exp(beta_K_sensi * tau_a) / lambda * g_beta_fun_gen(tau_c - tau_a)
  }
  
  # function g of x and the constant beta (Equation 6)
  g_beta_fun_gen <- function(x){(exp(beta_K_sensi * x) - 1) / beta_K_sensi}
  
  # function f of x and the constant beta (Equation 7)
  f_beta_fun_gen <- function(x){log(1 + beta_K_sensi * x) / beta_K_sensi}
  
  # normalise Ta, Tc and heating rate by Tk
  x <- x %>% # Calculate heating rate as lambda
    mutate(tau_a_low = ta_low / Tk,
           tau_a_high = ta_high / Tk,
           tau_c_low = tc_low / Tk,
           tau_c_high = tc_high / Tk,
           lambda = heating_rate / Tk,
           delta_tau_low = tau_c_low - tau_a_low, # calculate delta taus
           delta_tau_high = tau_c_high - tau_a_high)

  # Rescale lower temperature experiments
  x <- x %>% 
    mutate(delta_tr_low = pmap_dbl(list(tau_a = tau_a_low,
                                    tau_c = tau_c_low,
                                    lambda = lambda),
                               delta_tr_gen))
  
  # Rescale higher temperature experiments
  x <- x %>% 
    mutate(delta_tr_high = pmap_dbl(list(tau_a = tau_a_high,
                                     tau_c = tau_c_high,
                                     lambda = lambda),
                                delta_tr_gen))
  
  # Add predictions of high from low and conversely low from high
  x <- x %>% 
    mutate(activation = ev,
           delta_tau_high_hat = f_beta_fun_gen(exp(beta_K_sensi * (tau_a_low - tau_a_high)) * g_beta_fun_gen(delta_tau_low)), # estimate high from low
           delta_tau_low_hat = f_beta_fun_gen(exp(beta_K_sensi * (tau_a_high - tau_a_low)) * g_beta_fun_gen(delta_tau_high))) # estimate low from high

  return(x) # as list
}
```

The dataset is replicated for a vector of activation energies, then the `delta_tr_gen` function is applied to each element of the list using `mapply`.

```{r}
#| label: fig-sensitivity
#| fig-cap: "rescaled heating durations for the lower and higher acclimation temperatures calculated using values of activation energy between 0.2 and 1.2 eV. Observations (points) with a fitted linear model (solid line) fall along the identity line (dashed line)."

# Sequence of activation energies between 0.2 and 1.2
activation <- seq(0.2, 1.2, 0.2)
# Repeat ARR dataset over length of activation energies
ARR_list <- replicate(length(activation), ARR, simplify = FALSE)
# Rescale heating tolerances, merge to single data frame
ARR_list <- do.call(rbind, mapply(ARR_list, FUN = delta_tr_gen, ev = activation, SIMPLIFY = FALSE))

# Create plot of results
sensi_plot <- ggplot(data = ARR_list, 
                 mapping = aes(x = delta_tr_low, 
                               y = delta_tr_high)) + 
  geom_point(col = "grey") + 
  geom_smooth(method = "lm", se = FALSE, col = "black") + 
  geom_abline(slope = 1, intercept = 0, lty = 2, alpha = 0.6) +
  scale_x_continuous(expand = c(0.01,0), trans = "log10", labels = function(x) format(x, scientific = FALSE)) +
  scale_y_continuous(expand = c(0.01,0), trans = "log10", labels = function(x) format(x, scientific = FALSE)) +
  facet_wrap(.~activation, ncol=3, scales = "free") +
  labs(x = expression("Lower temperature rescaled heating duration"),
       y = expression("Higher temperature rescaled heating duration")) +
  theme_classic(base_size = 12)

sensi_plot
```

```{r}
#| label: tbl-sensitivity
#| tbl-cap: "R2 values of the regression between rescaled heating durations for the lower and higher acclimation temperatures calculated using a range of activation energies."

ARR_list %>% 
  group_by(activation) %>% 
  group_map(~ summary(lm(delta_tr_high ~ delta_tr_low, data = .x))$r.squared) %>% 
  unlist() %>% 
  data.frame(activation = activation, "R2" = .) %>% 
  knitr::kable(digits = 3, col.names = c("Activation energy (eV)", "R squared value"))
```

***

# Predicting heating tolerance for a new acclimation temperature

To test the importance of considering acclimation temperature and the non-linear temperature-dependence of biological rates in our analysis, we investigated a series of hypotheses representing assumptions made in our analysis from the simplest model to the full derivation. We estimated the heating tolerance at the higher acclimation temperature (an unknown value, $a2$) from the heating tolerance of the lower acclimation temperature (a known value, $a1$), via rescaling. We compared our estimations against the observed upper thermal tolerances. If our estimations matched our observed values, then our formulations were suitable. If not, then our assumptions are not valid and further derivations are required.

## Assuming heating tolerances are the same

The simplest formula to estimate a new heating tolerance from a new acclimation temperature is to assume that heating tolerances at the higher and lower acclimation temperature experiments are the same. For example, we estimated the heating tolerance at the higher acclimation temperature $\Delta \tau (\tau_{a2})$, from the lower acclimation temperature, $\Delta \tau (\tau_{a1})$.

$$
\Delta \tau (\tau_{a2}) \approx \Delta \tau (\tau_{a1})
$$ {#eq-S4}

```{r}
#| label: fig-predH0
#| fig-cap: "Observed vs estimated heating tolerance for the higher acclimation temperature (°C) assuming heating tolerance is the same as the lower acclimation temperature. Predictions were made for each species (points). Predictions should fall along a 1:1 identity line (dashed line) if assuming that the heating tolerance of the higher temperature is the same as at the lower temperature is accurate. The solid line is an ordinary least-squares regression."

pred1 <- ggplot(data = ARR,
       mapping = aes(y = delta_tau_low * Tk,
                     x = delta_tau_high * Tk)) + 
  geom_point() + 
  geom_abline(intercept = 0, slope = 1, lty = 2, alpha = 0.6) + 
  geom_smooth(method = "lm", se = FALSE, col = "darkgrey") + 
  labs(y = expression("Estimated heating tolerance (\u00B0C)"),
       x = expression("Observed heating tolerance (\u00B0C)")) + 
  scale_x_continuous(expand = c(0,0), limits = c(0, 40)) +
  scale_y_continuous(expand = c(0,0), limits = c(0, 40))

pred1
```

```{r}
#| code-fold: true
# high from low R2
pred_H0 <- lm(delta_tau_low ~  delta_tau_high, 
                   data = ARR) %>% 
  summary(.) %>%
  .$r.squared %>% 
  round(., 4) * 100
```

Assuming heating tolerance is the same at both acclimation temperatures overestimated the heating tolerance at the higher temperature (@eq-S4); points fall above the identity line (@fig-predH0). The $R^2$ is `r pred_H0`%.

## Correcting for acclimation temperature difference

Next, we can account for the difference in acclimation temperatures by rescaling by the acclimation temperature via $e^{\beta (\tau_{a1} - \tau_{a2})}$:

$$
\Delta \tau (\tau_{a2}) \approx \Delta \tau (\tau_{a1}) e^{\beta (\tau_{a1} - \tau_{a2})}
$$ {#eq-S5}

```{r}
#| label: fig-H1
#| fig-cap: "Observed vs estimated heating tolerance for the higher acclimation temperature (°C) accounting for the change in acclimation temperature. Predictions were made for each species (points). Predictions should fall along a 1:1 identity line (dashed line) if the model is accurate. The solid line is an ordinary least-squares regression."

pred2 <- pred1 + aes(y = (delta_tau_low * exp(beta_K * (tau_a_low - tau_a_high))) * Tk) +
  labs(y = expression("Estimated heating tolerance (\u00B0C)")) 
pred2
```

```{r}
#| code-fold: true
# high from low R2
pred_H1 <- lm(((delta_tau_low * exp(beta_K * (tau_a_low - tau_a_high)))) ~  delta_tau_high, 
                   data = ARR) %>% 
  summary(.) %>%
  .$r.squared %>% 
  round(., 4) * 100
```

Accounting for the difference in acclimation temperature when rescaling heating tolerances underestimates thermal tolerances for the higher acclimation temperature (@eq-S5); points fall below the identity line (@fig-H1). The $R^2$ is `r pred_H1`%.

## Account for difference in acclimation temperature and change in temperature

Finally, we can derive a more precise formulation that fully accounts for the change to heating tolerance that occurs during the experiment as the temperature is increased using Equation 8 in the main text (below). 

$$
\Delta \tau (\tau_{a2}) \approx f_\beta \big(e^{\beta (\tau_{a1} - \tau_{a2})} g_\beta(\Delta \tau(\tau_{a1})) \big)
$$
To test the consistency of the rescaling estimations between the two acclimation temperatures, we can estimate the heating tolerance at the lower acclimation temperature from the heating tolerance at the higher acclimation temperature (@eq-S6). Equation 8 in the main text and @eq-S6 converts normalised heating tolerance at one acclimation temperature into rescaled heating duration (via $g_\beta$), corrects for the difference in acclimation temperature along the non-linear temperature-dependence of biological rates (via $e^{\beta\tau}$), and converts rescaled and biological rate-corrected heating tolerance into normalised heating tolerance at the other acclimation temperature (via $f_\beta$). 

$$
\Delta \tau (\tau_{a1}) \approx f_\beta \big(e^{\beta (\tau_{a2} - \tau_{a1})} g_\beta(\Delta \tau(\tau_{a2})) \big)
$$ {#eq-S6}

```{r}
# Add estimations of high from low and conversely low from high acclimation temperature (Equations 8 & S6)
ARR <- ARR %>% 
  mutate(delta_tau_high_hat = f_beta_fun(exp(beta_K * (tau_a_low - tau_a_high)) * g_beta_fun(delta_tau_low)), # estimate high from low (Equation 8 in the main text)
         delta_tau_low_hat = f_beta_fun(exp(beta_K * (tau_a_high - tau_a_low)) * g_beta_fun(delta_tau_high))) # estimate low from high (Equation S6)
```

## Figure 3

Generate Figure 3 in the main text.

```{r}
#| column: page
#| layout-ncol: 2
#| fig-subcap: 
#|  - "Predicting heating tolerance of the higher acclimation temperature from the lower acclimation temperature. Figure 3A in main text."
#|  - "Predicting heating tolerance of the lower acclimation temperature from the higher acclimation temperature. Figure 3B in main text."

pred3 <- ggplot(data = ARR,
       mapping = aes(y = delta_tau_high_hat * Tk,
                     x = delta_tau_high * Tk)) + 
  geom_point() + 
  geom_abline(intercept = 0, slope = 1, lty = 2, alpha = 0.6) + 
  geom_smooth(method = "lm", se = FALSE, col = "darkgrey") + 
  labs(y = expression("Estimated heating tolerance (\u00B0C)"),
       x = expression("Observed heating tolerance (\u00B0C)")) + 
  scale_x_continuous(expand = c(0,0), limits = c(0, 40)) +
  scale_y_continuous(expand = c(0,0), limits = c(0, 40)) 

pred3

pred4 <- ggplot(data = ARR,
                mapping =aes(y = delta_tau_low_hat * Tk,
                             x = delta_tau_low * Tk)) + 
  geom_point() + 
  geom_abline(intercept = 0, slope = 1, lty = 2, alpha = 0.6) + 
  geom_smooth(method = "lm", se = FALSE, col = "darkgrey") + 
  labs(y = expression("Estimated heating tolerance (\u00B0C)"),
       x = expression("Observed heating tolerance (\u00B0C)")) + 
  scale_x_continuous(expand = c(0,0), limits = c(0, 40)) +
  scale_y_continuous(expand = c(0,0), limits = c(0, 40))
pred4
```

```{r pred3-lm}
#| code-fold: true
# Calculate R2
pred_high_low <- lm(delta_tau_high_hat ~ delta_tau_high, 
                   # offset = delta_tau_high_hat, 
                   data = ARR)

pred_low_high <- lm(delta_tau_low_hat ~ delta_tau_low, 
                   # offset = delta_tau_low_hat, 
                   data = ARR)
```

The $R^2$ of predicting the thermal tolerance of the higher acclimation temperature from the lower acclimation temperature is `r round(summary(pred_high_low)$r.squared, 4)*100`%, and the $R^2$ of predicting the thermal tolerance of the lower acclimation temperature from the higher acclimation temperature is `r round(summary(pred_low_high)$r.squared, 4)*100`%.

***

# The effect of heating rate on rescaled heating duration

```{r varying-rates}
# Import and prepare Morley et al 2016
varying_rates <- read.csv("data/Morley_et_al_2016.csv") %>% 
  rename(heating_rate = 8,
         ta = 9,
         tc = 10) %>% 
   mutate(Genus = ifelse(Species == "borchgrevinki", "Pagothenia", genus)) %>% # Synonym Trematomus Pagothenia Pagothenia in ARR dataset
  mutate(Name = paste(Genus, Species), 
         experiment = "variable",
         lambda = heating_rate / Tk) %>%
  # Update phyla names
  mutate(Phyla = case_when(phyla == "mollusc" ~ "Mollusca",
                           phyla == "Fish" ~ "Chordata",
                           phyla == "Ascidians" ~ "Chordata",
                           phyla == "Crustacean" ~ "Arthropoda",
                           TRUE ~ phyla)) %>%
  group_by(Name) %>%
  distinct() %>% # remove duplicated rows
  filter(n()>1) %>% # remove species with only 1 observation
  ungroup()

# Add Acclimation Response Ratio dataset to Morley et al 2016
species_data <- ARR %>%
  # Transform the ARR dataset to long format
  pivot_longer(cols = starts_with(c("ta_", "tc_")),
               names_to = c(".value", "assay"),
               names_sep = "_") %>%
  mutate(experiment = "constant") %>% 
  # Join datasets and get occurences of species in each dataset
  full_join(.,
            varying_rates, 
            by = c("Genus", "Species", "Name", "experiment", "ta", "tc", "lambda"),
            suffix = c("_constant", "_variable")) %>% 
  # Normalise temperature
  mutate(tau_a = ta / Tk, 
         tau_c = tc / Tk,
         delta_tau = tau_c - tau_a) %>% 
  group_by(Name) %>% 
  mutate(occurrence = length(unique(experiment))) %>%
  ungroup() %>% 
  # Keep species with more than 1 heating rate from either dataset
  filter(experiment == "variable" | occurrence == 2) %>% 
  # Calculate rescaled heating duration (Equation 8)
  mutate(delta_tr = pmap_dbl(list(tau_a = tau_a,
                                  tau_c = tau_c,
                                  lambda = lambda),
                             delta_tr_fun))
```

To examine the relationship between heating rate and rescaled heating duration further, we tested a series of hypotheses describing how heating rate was expected to affect the rescaling of heating tolerances. Since the Acclimation Response Ratio dataset does not contain observations made under different heating rates from the same starting temperature for a species, we used a second dataset containing upper thermal tolerances measured from the same starting temperature under different heating rates ($\lambda$) [@Morley2016]. Doing so allowed us to examine the effect of varying heating rate while keeping acclimation or starting temperature the same. We removed species with only one observation were removed (n = 3), resulting in a dataset with `r nrow(varying_rates)` observations from `r length(unique(varying_rates$Phyla))` phyla. *Trematomus borchgrevinki* was renamed to *Pagothenia borchgrevinki* because *Pagothenia* is the accepted genus and is also used in constant heating rates dataset. We combined this dataset (`varying_rates`) with data from the constant heating rate experiments for species that also had variable heating rate data with them (`r nrow(species_data)` observations from `r length(unique(species_data$Species))` species, called `species_data`). The datasets are identified by `experiment` (`variable` or `constant`) and `occurrence` and some species occurred in both datasets.

***

## Testing a null effect of heating rates on rescaled heating duration

First we tested a null hypothesis that rescaled heating duration does not depend on heating rate. For any heating rate:

$$
\Delta \tau (\lambda)= f_\beta (e^{-\beta \tau_{a}} \lambda \Delta t_r(\lambda)) 
$$ {#eq-S7}

If the null hypothesis is supported (@eq-S7), then $\Delta t_r (\lambda) = \Delta t_r (0)$ and thus @eq-S7 becomes Equation 9 in the main text (below).

$$
\Delta \tau (\lambda) = f_\beta (e^{-\beta \tau_{a}} \lambda \Delta t_r (0))
$$

which is a logarithmic, temperature-dependent relationship between observed heating tolerance and heating rate. Equation 9 can be tested by conducting ramping assays starting at the same acclimation temperature but using different heating rates. From these data we can infer $\Delta t_r (0)$ from the experiment with the slowest heating rate $\lambda_{min}$. We should then have that

$$
\Delta \tau (\lambda) = f_\beta (\hat{\lambda}), \ \text{where} \ \hat{\lambda} = \frac{\lambda}{\lambda_{min}} g_\beta (\Delta \tau (\lambda_{min}))
$$ {#eq-S8}

In a plot of normalised heating tolerance ($\Delta \tau$) against the transformed variable $f_\beta (\hat{\lambda})$, the null hypothesis (@eq-S8) is supported if the points fall along the identity (1:1) line. If points systematically fall below the 1:1 line, then the null hypothesis is not supported (Figure 4A in main text).

```{r}
# Calculate ratios of Equation S8 and Figure 4A
rate_ratios <- species_data %>% 
  # Group by name
  group_by(Name) %>% 
  # For each species create all combinations of heating rate with covariates
  group_modify(~expand_grid(.x, 
                            select(.x, lambda, delta_tr, delta_tau, tau_a),
  # Add numbered suffix to all columns; unique for duplications
                            .name_repair = function (x) ave(x, x, FUN = function(i) str_c(i, seq_along(i))))) %>%
  # Calculate ratio of rescaled duration and heating rate (Equation 19)
  mutate(lambda_ratio = lambda2/lambda1,
         t_ratio = delta_tr2/delta_tr1) %>%
  # Calculate scaling coefficient gamma for each species (Equation 19)
  group_modify(function(x, y){
    x$gg <- abs(as.numeric(coef(lm(log(t_ratio) ~ log(lambda_ratio), x))[2]))
    return(x)}) %>% 
  distinct() %>% # Remove duplications created by expand grid
  ungroup() %>% 
  # Calculate estimated normalised heating tolerance at the second acclimation temperature, corrected for heating rate (Equation 20)
  mutate(delta_tau2_hat = f_beta_fun(exp(beta_K * (tau_a1-tau_a2)) * (lambda_ratio ^ (1 - gg)) * g_beta_fun(delta_tau1)),
         delta_tau2_hat_inter = f_beta_fun(exp(beta_K * (tau_a1-tau_a2)) * (lambda_ratio ^ (1 - 0.7656548)) * g_beta_fun(delta_tau1)))

# Get interspecific gamma
gamma <- abs(coef(glm(log(t_ratio) ~ log(lambda_ratio), data = rate_ratios))[2])
```

## Figure 4A

We used the slowest heating rate of each species to test whether heating rate had no effect on rescaled heating duration and thus estimated heating tolerance. The null hypothesis was not supported because points systematically fall below the 1:1 line (Figure 4A in main text).

```{r}
#| fig-cap: "Estimateed heating tolerance versus observed heating tolerance for species with varying heating rates (107 observations). Colours are species (n = 37). Figure 4A in main text."

g_departure <- species_data %>%
  filter(experiment == "variable") %>% 
  group_by(Name) %>% 
  mutate(lambda_min = min(lambda),
         delta_tau_min = delta_tau[which.min(lambda)]) %>% 
  ungroup() %>% 
  ggplot(mapping = aes(x = f_beta_fun(lambda / lambda_min * g_beta_fun(delta_tau_min)) * Tk,
                       y = delta_tau * Tk,
                       col = Name)) + 
  geom_point() + 
  geom_line(alpha = 0.5) +
  geom_abline(slope = 1, intercept = 0, lty = 2, alpha = 0.6) +
  labs(x = expression("Estimated heating tolerance (\u00B0C)"), 
       y = expression("Observed heating tolerance (\u00B0C)")) +
  scale_colour_viridis_d(option = "C", end = 0.9) +
  scale_x_continuous(expand = c(0,0), limits = c(0, 100)) +
  scale_y_continuous(expand = c(0,0), limits = c(0, 25)) +
  theme(legend.position="none", plot.margin = margin(5.5, 7, 5.5, 5.5, unit = "pt"))

g_departure
```


## Figure 4B

```{r}
#| fig-cap: "Interspecific scaling of rescaled heating duration with heating rate. Variation within each species is shown by the fitted lines between points (129 observations). The dashed identity line is the expected relationship following the null hypothesis. Axes are Log10 transformed. Colours are species (n = 37). Figure 4B in main text."

g_scaling <- ggplot(data = rate_ratios,
                    mapping = aes(x = lambda_ratio, 
                                  y = t_ratio, 
                                  group = Name,
                                  col = Name)) + 
  geom_line(stat ="smooth", method = "lm", alpha = 0.5) +
  geom_point() + 
  geom_abline(slope = -1, intercept = 0, lty = 2, alpha = 0.6) +
  geom_smooth(aes(group = 1), method = "lm", se = FALSE, col = 1) +
 scale_x_log10(
   breaks = scales::trans_breaks("log10", function(x) 10^x),
   labels = scales::trans_format("log10", scales::math_format(10^.x))
 ) +
 scale_y_log10(
   breaks = scales::trans_breaks("log10", function(x) 10^x),
   labels = scales::trans_format("log10", scales::math_format(10^.x))
 ) +
  ggtitle(paste0("slope = ", 
                 round(gamma, 2))) +
  labs(x = "Heating rate ratio", y = "Rescaled heating duration ratio") +
  scale_colour_viridis_d(option = "C", end = 0.9) +
  theme(legend.position="none")

g_scaling
```

To characterise the effect of heating rate on rescaled heating duration, we plot the ratio of rescaled heating duration for a paired observation within a species with the corresponding ratio for paired heating rate. The relationship between the ratio of rescaled heating duration and the ratio of heating rate is described by an allometric scaling equation in the form $\Delta t_r (\lambda) \sim c \lambda^{- \gamma}$ where $c$ depends only on the experimental protocol and the species-specific scaling exponent, $\gamma$. Points should fall along the identity line if heating rate does not affect rescaled heating duration (i.e., a scaling exponent of -1).

Change in rescaled heating duration, and thus heating tolerance, scales with change in heating rate according to an allometric scaling equation. There is some species-specific variation in the slope of the regression $\gamma$ (colours). We can calculate an interspecific scaling exponent of $\gamma =$ `r round(gamma, 2)` calculated from a linear regression of ratios of heating rate and ratios of rescaled heating duration on Log~10~ scales (Figure 4B in main text).

***

## Quantifying the effect of heating rates on rescaled heating duration

Since higher heating rates reduces rescaled heating duration following a phenomenological power law, then the relationship between two experiments done at two heating rates, $\lambda_1$ and $\lambda_2$, regardless of the starting temperature, can be described by:

$$
\frac{\lambda_2 \Delta t_r (\lambda_2)}{\lambda_1 \Delta t_r (\lambda_1)} \approx \bigg(\frac{\lambda_2}{\lambda_1}\bigg)^{1-\gamma}
$$ {#eq-S9}

and therefore that from one heating tolerance $\Delta \tau_1$ we should be able to estimate any other $\Delta \tau_2$. Using @eq-S9 we can deduce $\Delta t_r (\lambda_2)$ and from Equations 6 and 7 leads to Equation 10 in the main text:

$$
\Delta\tau_2 = f_{\beta}\Bigg(e^{\beta(\tau_{a1}-\tau_{a2})}\bigg(\frac{\lambda_2}{\lambda_1}\bigg)^{1-\gamma}g_{\beta}\big(\Delta\tau_1\big)\Bigg)
$$

That is, we can estimate one unknown heating tolerance $\Delta\tau_2$ from another known value $\Delta\tau_1$ using Equation 10 in the main text (above) which incorporates the correction for the effect of heating rate on rescaled heating duration using a species-specific scaling exponent ($\gamma$). We use species-specific scaling exponents in subsequent calculations for precision rather than the interspecific scaling exponent. Doing so, we see strong concordance of the observed heating tolerance (`delta_tau2`) and the estimated value (`delta_tau2_hat`).

## Figure 4C

```{r}
#| fig-cap: "Estimated heating tolerance corrected using species-specific scaling exponents for the allometric relationship between rescaled heating duration and heating rate. Colours are species. Figure 4C in main text."

g_estimate_tau <- rate_ratios %>% 
  filter(t_ratio != 1 & lambda_ratio != 1) %>% # remove trivial pairs where it is the lowest heating rate
  ggplot(mapping = aes(x = delta_tau2_hat * Tk, 
                       y = delta_tau2 * Tk,
                       col = Name)) + 
  geom_abline(slope = 1, intercept = 0, lty = 2, alpha = 0.6) +
  geom_point() + 
  geom_smooth(method = "lm", se = FALSE, col = 1) + 
  labs(x = expression("Estimated heating tolerance (\u00B0C)"), 
       y = expression("Observed heating tolerance (\u00B0C)")) +
  scale_colour_viridis_d(option = "C", end = 0.9) +
  scale_x_continuous(expand = c(0,0), limits = c(0, 30)) +
  scale_y_continuous(expand = c(0,0), limits = c(0, 30)) +
  theme(legend.position="none")

g_estimate_tau
```

```{r}
# R2 of the scaling relationship using species-specific values of gamma
species_gamma <- rate_ratios %>% 
   filter(t_ratio != 1 & lambda_ratio != 1) %>% 
  lm(delta_tau2_hat ~  delta_tau2, .) %>% 
  summary(.) %>%
  .$r.squared %>% 
  round(., 4) * 100
```

The $R^2$ is `r species_gamma`%.

## Interspecific scaling exponent of $\gamma$

```{r}
# R2 of the scaling relationship using the interspecific value of gamma
interspecific_gamma <- rate_ratios %>% 
   filter(t_ratio != 1 & lambda_ratio != 1) %>% 
  lm(delta_tau2_hat_inter ~  delta_tau2, .) %>% 
  summary(.) %>%
  .$r.squared %>% 
  round(., 4) * 100
```

We plotted the same as Figure 4C but using the interspecific value of `r round(gamma, 2)` for $\gamma$. Doing so increases our estimation error for a species (@fig-gamma-inter). The $R^2$ is `r interspecific_gamma`%.

```{r}
#| label: fig-gamma-inter
#| fig-cap: "Estimated heating tolerances calculated using a value of 0.76 for gamma, representing an interspecific parameter. Colours are species."

rate_ratios %>% 
   filter(t_ratio != 1 & lambda_ratio != 1) %>% # remove trivial pairs where it is the lowest heating rate
  ggplot(mapping = aes(x = delta_tau2_hat_inter * Tk, 
                       y = delta_tau2 * Tk,
                       col = Name)) + 
  geom_abline(slope = 1, intercept = 0, lty = 2, alpha = 0.6) +
  geom_point() + 
  geom_smooth(method = "lm", se = FALSE, col = 1) + 
  scale_x_continuous(expand = c(0,0), limits = c(0, 30)) +
  scale_y_continuous(expand = c(0,0), limits = c(0, 30)) +
  labs(x = expression("Estimated heating tolerance (\u00B0C)"), 
       y = expression("Observed heating tolerance (\u00B0C)")) +
  scale_colour_viridis_d(option = "C", end = 0.9) +
  theme(legend.position="none")
```

***

# Generalised graphs of rescaled heating durations

We visualised the generalised relationship described by Equation 10 in the main text, using median values of heating rate, upper thermal tolerance and acclimation temperature across all species and the interspecific scaling exponent, $\gamma =$ `r round(gamma, 2)`.

```{r}
#| label: fig-gen
#| fig-cap: "Generalised graph of estimated heating tolerance by acclimation temperature and heating rate (colours)."

sp_heat_rate = median(ARR$heating_rate)
sp_ta = median(ARR$ta_low)
sp_tc = median(ARR$tc_low)

heating_rate <- round(sort(c(10^(seq(log10(0.004), log10(60), length.out = 14)), 0.015, 1, 0.042, 18)), 3) # on log10 scale for equidistant points
heating_rate <- heating_rate[!heating_rate %in% c(0.018, 0.037, 13.667)] # neaten lines
ta <- seq(5, 40, 1) # range of acclimation temperatures
pal_cols <- viridisLite::viridis(n = length(heating_rate), end = 0.9) # colour palette for points

gen_ecto <- expand_grid(heating_rate, ta) %>% 
  mutate(sp_lambda = sp_heat_rate / Tk,
         sp_tau_a = sp_ta / Tk,
         sp_tau_c = sp_tc / Tk,
         sp_delta_tau = sp_tau_c - sp_tau_a,
         tau_a = ta / Tk, 
         delta_tau_a =  sp_tau_a - tau_a,
         lambda = heating_rate / Tk,
         lambda_ratio = lambda/sp_lambda)  %>% 
  mutate(delta_tau_hat =  f_beta_fun(exp(beta_K * delta_tau_a) * (lambda_ratio ^ (1 - gamma)) * g_beta_fun(sp_delta_tau))) %>% 
  ggplot() + 
  geom_line(aes(ta, delta_tau_hat * Tk, group = heating_rate, col = heating_rate)) +
  labs(x = expression("Acclimation temperature (\u00B0C)"), 
       y = expression("Expected heating tolerance (\u00B0C)")) +
  scale_colour_viridis_c(name = expression("Heating rate (\u00B0C h" ^-1 * ")"), end = 0.9, trans = "log10", labels = function(x) signif(x)) 

gen_ecto
```

## Figure 5

@fig-gen can also be visualised as estimated upper thermal tolerance limit $T_c$ for any acclimation temperature $T_a$ .

```{r}
#| fig-cap: "Generalised graph of estimated upper thermal tolerance limit by acclimation temperature and heating rate (colours). Figure 5 in the main text."

fig5 <- expand_grid(heating_rate, ta) %>% 
  mutate(sp_lambda = sp_heat_rate / Tk,
         sp_tau_a = sp_ta / Tk,
         sp_tau_c = sp_tc / Tk,
         sp_delta_tau = sp_tau_c - sp_tau_a,
         tau_a = ta / Tk, 
         delta_tau_a =  sp_tau_a - tau_a,
         lambda = heating_rate / Tk,
         lambda_ratio = lambda/sp_lambda)  %>% 
  mutate(delta_tau_hat =  f_beta_fun(exp(beta_K * delta_tau_a) * (lambda_ratio ^ (1 - gamma)) * g_beta_fun(sp_delta_tau))) %>% 
  ggplot() + 
  geom_line(aes(ta, delta_tau_hat * Tk + ta, group = heating_rate, col = heating_rate)) +
  labs(x = expression("Acclimation temperature (\u00B0C)"), 
       y = expression("Upper thermal tolerance limit (\u00B0C)")) +
  scale_colour_viridis_c(name = expression("Heating rate (\u00B0C h" ^-1 * ")"),
                         trans = "log10",
                         end = 0.9,
                         labels = function(x) signif(x)) 

fig5
```

***

# Table of equations

| Equation | Description |
|:------:|:------------|
| 1 | Heating tolerance; range of temperatures between acclimation temperature and upper thermal tolerance  | 
| 2 | Elapsed duration of a heating assay between the start ($t_a$) and end ($t_c$) time points |
| @eq-S1 | Describing the relationship between two heating durations |
| @eq-S2 | Describing the integration of the exponential temperature-dependence of biological rates between the start and end of the ramping assay |
| @eq-betaS3 | Deriving the $\beta$ coefficient |
| 3 | Approximating the temperature-dependence of biological rates with the $\beta$ coefficient, @eq-betaS3 |
| 4 | Simplified form of @eq-S2 using @eq-betaS3 describing the same relationship |
| 5 | Simplified form of Equation 4 describing the same relationship and including a constant heating rate ($\lambda$) |
| 6 | The integration of Equation 5 to calculate rescaled heating duration from heating tolerance on a normalised temperature scale |
| 7 | The inverse of Equation 6 to calculate heating tolerance on a normalised temperature scale from rescaled heating duration |
| @eq-S4 | The simplest hypothesis for how one known heating tolerance can be used to estimate an unknown heating tolerance at another acclimation temperature, assuming rescaled heating durations are the same |
| @eq-S5 | An alternative hypothesis for how one known heating tolerance can be used to estimate an unknown heating tolerance at another acclimation temperature, accounting for differences in acclimation temperature |
| 8 | A fully derived hypothesis for how one known heating tolerance can be used to estimate an unknown heating tolerance at another acclimation temperature, accounting for differences in acclimation temperature and the integration of the exponential temperature-dependence of biological rates. Used to estimate thermal tolerance at the higher acclimation temperature from the lower acclimation temperature |
| @eq-S6 | The same as Equation 8 to estimate thermal tolerance at the lower acclimation temperature from the higher acclimation temperature |
| @eq-S7 | General expression describing that heating rate has no effect on rescaled heating duration |
| 9 | Extension of @eq-S7 describing the null hypothesis that heating rate has no effect on rescaled heating duration |
| @eq-S8 | Extension of Equation 9 describing the null hypothesis that heating rate has no effect on rescaled heating duration based on comparing the ratio of the slowest heating rate with all other rates measured for a species |
| @eq-S9 | General description of the power scaling relationship between two assays measured using two heating rates |

: All numbered equations in supplementary (denoted Equation S) and main text in the order presented. {#tbl-equations}

***

# Manuscript Figures

```{r figs, echo=T, eval = F}
#| code-fold: true
# rescaled graphs
legend <- get_legend(heating_graph + theme(legend.box.margin = margin(0, 0, 0, 0)))

p2 <- plot_grid(heating_graph + theme(legend.position="none"),
                rescaled_duration + theme(legend.position="none"),
                # rescaled_tolerances + theme(legend.position="none"),
                labels = "AUTO", nrow = 1, align = "hv", hjust = -1, label_size = 10)

fig2 <- plot_grid(p2, legend, rel_widths =c(3, 0.5))
fig2

# estimation graphs
pred_high_low <- pred3 + aes(col = heating_rate) + 
  scale_x_continuous(expand = c(0,0), limits = c(0, 40)) +
  scale_y_continuous(expand = c(0,0), limits = c(0, 40)) +
  geom_smooth(method = "lm", se = FALSE, col = 1) +
  scale_colour_viridis_c(name = expression("Heating rate (\u00B0C h" ^-1 * ")"), end = 0.9, trans = "log10", labels = function(x) signif(x)) +
  theme(legend.position="none")

pred_low_high <- pred4 + aes(col = heating_rate) + 
  scale_x_continuous(expand = c(0,0), limits = c(0, 40)) +
  scale_y_continuous(expand = c(0,0), limits = c(0, 40)) +
  geom_smooth(method = "lm", se = FALSE, col = 1) +
  scale_colour_viridis_c(name = expression("Heating rate (\u00B0C h" ^-1 * ")"), end = 0.9, trans = "log10", labels = function(x) signif(x)) +
  theme(legend.position="none")

p3 <- plot_grid(pred_high_low, pred_low_high, labels = "AUTO", nrow = 1, align = "h", hjust = -1, label_size = 10)
fig3 <- plot_grid(p3, legend, rel_widths =c(3, 0.5))
fig3

# Heating rate effect
fig4 <- plot_grid(g_departure, g_scaling, g_estimate_tau, labels = "AUTO", nrow = 1, align = "h", hjust = -1, label_size = 10)
fig4
```

Figures saved as TIFF. Resulting dataset containing all analyses (`ARR`) are saved as CSV.

```{r save, eval = F}
#| code-fold: true
journal <- "JAnim"
dir.create(paste0("../figures/", journal, "/"))

for(i in c("tiff", "jpeg")){
save_plot(plot = fig2, filename = paste0("../figures/", journal, "/fig2.", i), dpi = 300, base_width = 10, bg = "white")
save_plot(plot = fig3, filename = paste0("../figures/", journal, "/fig3.", i), dpi = 300, base_width = 10, bg = "white")
save_plot(plot = fig4, filename = paste0("../figures/", journal, "/fig4.", i), dpi = 300, base_width = 8, base_height = 3, bg = "white")
save_plot(plot = fig5, filename = paste0("../figures/", journal, "/fig5.", i), dpi = 300, base_width = 5, bg = "white")
}

# Supplementary
save_plot(plot = sensi_plot, filename = paste0("../figures/", journal, "/figS1.jpeg"), dpi = 300, base_width = 10, base_height = 5, bg = "white")
# save_plot(plot = rescaled_tolerances, filename = paste0("../figures/", journal, "/figS2.jpeg"), dpi = 300, bg = "white")
save_plot(plot = pred1, filename = paste0("../figures/", journal, "/figS3.jpeg"), dpi = 300, bg = "white")
save_plot(plot = pred2, filename = paste0("../figures/", journal, "/figS4.jpeg"), dpi = 300, bg = "white")

# data
write.csv(ARR, file = "output/rescaled_heating_tolerances_data.csv", row.names = FALSE)
```

***

# Session information {#sec-session}
```{r, echo=FALSE}
sessionInfo()
```

***

# References
```
