# Supplementary material for: Heat limits scale with metabolism in ectothermic animals
Source: J Anim Ecol. 2025 May 12;94(6):1307–16. doi: 10.1111/1365-2656.70042 (PMC12134445; doi:10.1111/1365-2656.70042)
Supplement: Supplementary file 2 — Supplementary File S2. Expanded mathematical explanation of our derivation of the exponential approximation of the Arrhenius form. [file JANE-94-1307-s002.pdf]

Code

Derivation of the exponential approximation for the Arrhenius equation

Define symbols

Symbol	Explanation
y	metabolic rate
T	Temperature in Kelvin
T_K	Kelvin equivalent of $0^{\circ}\mathrm{C}$
T°	Temperature in C
E_a	Activation energy in eV
k	Boltzmann's constant $\approx 8.6 \times 10^{-5}$ in eV/K
A	Scaling coefficient

The Arrhenius equation is defined as a function of degrees Kelvin

$$y = Ae^{-\frac{E_a}{kT}}$$

Some re-writing noting that $T = T_K + T^{\circ}$ yields

$$y = Ae^{-\frac{E_a}{kT}} = Ae^{-\frac{E_a}{k}\frac{1}{T}} = Ae^{-(\frac{E_a}{k})(\frac{1}{T_K+T^{\circ}})}$$

we want to take advantage of the fact that by Taylor series expansion, since $|\frac{T^{\circ}}{T_K}| < 1$ we have

$$\frac{T_K}{T_K + T^{\circ}} \approx 1 - \frac{T^{\circ}}{T_K} + \frac{T^{\circ 2}}{T_K^2} - \frac{T^{\circ 3}}{T_K^3} \dots$$

For biologically relevant temperatures in the range -10C to 50C, the first portion of the Taylor expansion is sufficiently accurate. Compare corresponding values of the left hand side of (1.0380228, 0.8452012) with right hand side approximations of (1.0366300, 0.8168498) for the first order approximation and (1.0379718 0.8503938) for the second order.

And so taking the first order approximation

$$\frac{T_K}{T_K + T^{\circ}} \approx 1 - \frac{T^{\circ}}{T_K}$$

we write

$$y = Ae^{-(\frac{E_a}{k})(\frac{1}{T_K})(\frac{T_K}{T_K+T^{\circ}})}$$

meaning we can approximate it as

$$y \approx Ae^{-(\frac{E_a}{k})(\frac{1}{T_K})(1-\frac{T^{\circ}}{T_K})}$$

we then define

$$\beta = \frac{E_a}{kT_K}$$

and our rescaled dimensionless temperature

$$\tau = \frac{T^{\circ}}{T_K}$$

so that we can write

$$y \approx Ae^{-(\beta)(1-\tau)} = Ae^{\beta(\tau-1)}$$

$$y \approx Ae^{-\beta}e^{\beta\tau}$$

We then collect the coefficients into a new scaling parameter

$$A_1 = Ae^{-\beta}$$

allowing us to finally write

$$y \approx A_1 e^{\beta\tau}$$

which gives the approximation that at temperatures close to biologically relevant ones with the rescaling to consider $\tau = T^{\circ}/T_K$ the Arrenhius function resembles closely a simple exponential.

$$Arr(T(\tau)) \propto e^{\beta\tau}$$
